# Impact of signs and symptoms of dry eye disease on health-related quality of life: a cross-sectional population study among older adults

**DOI:** 10.1007/s11136-025-03907-0

**Published:** 2025-01-28

**Authors:** Ulla Aapola, Paula Mosallaei, Janika Nättinen, Ilona Suurkuukka, Jaakko Tuomilehto, Sirkka Keinänen-Kiukaanniemi, Jouko Saramies, Hannu Uusitalo

**Affiliations:** 1 Eye and Vision Research, Faculty of Medicine and Health Technology, Tampere University, Tampere, Finland; 2 Finnish Register of Visual Impairment, Helsinki, Finland; 3 Fimea, Finnish Medicines Agency, Helsinki, Finland; 4 South Karelia Social and Health Care District, Lappeenranta, Finland; 5 Population Health Unit, Finnish Institute for Health and Welfare, Helsinki, Finland; 6 Center for Life Course Health Research, University of Oulu, Oulu, Finland; 7 Tays Eye Centre, Tampere University Hospital, Wellbeing Services County of Pirkanmaa, Tampere, Finland

**Keywords:** Ocular surface health, Dry eye disease, Quality of life, Sex differences, SF-36, 15D

## Abstract

**Purpose:**

To assess the relationship between quality of life (QoL) and ocular surface health within a Finnish population-based cohort.

**Methods:**

A cross-sectional study involved 601 individuals born between the years 1933–1956. Ocular surface health and dry eye disease (DED) were clinically evaluated using several diagnostic tests. Participants completed the Ocular Surface Disease Index (OSDI), QoL assessment with the 15D and Medical Outcomes Study 36-Item Short Form Health Survey (SF-36), and the Beck’s Depression Inventory (BDI-II) questionnaires. Various statistical methods were employed to explore the associations between QoL, ocular surface health, and sex disparities.

**Results:**

DED had negative impact on QoL in all participants, and especially in women. Adjusted for comorbidities, DED doubled the odds of worse health-related QoL (15D: OR = 2.31 [95% CI: 1.24–4.31, *p* < 0.01]) and mental health (SF-36 MCS and BDI-II: OR = 2.08 [95% CI: 1.04–4.16, *p* < 0.05]). Noninvasive tear break-up time (NIBUT) correlated with all QoL scores. In women, the most significant clinical signs correlating with low QoL were NIBUT (15D: *r* = 0.20, *p* = 0.002; SF-36 MCS: *r* = 0.18, *p* = 0.026), and conjunctival staining (15D: *r*=-0.19, *p* = 0.004; BDI-II: *r* = 0.27, *p* < 0.001), whereas in men, blepharitis correlated with depression score (BDI-II: *r* = 0.20, *p* = 0.036). High OSDI was associated with worse QoL in women, but not in men.

**Conclusion:**

This first population-based study assessing general QoL data with objective clinical measures of DED indicated that among elderly population, both symptoms and signs of DED independently impacted different aspects of QoL. In addition, significant sex-differences in these associations were observed and should be considered both in research settings and when assessing and treating people with DED.

**Supplementary Information:**

The online version contains supplementary material available at 10.1007/s11136-025-03907-0.

## Introduction

A healthy ocular surface is essential for clear vision, comfort, and prevention of various eye disorders. The ocular surface is covered by a thin fluid layer, the tear film, which lubricates, protects, and supplies nutrients and optical clarity for the ocular surface [1]. Imbalances in tear film homeostasis can lead to ocular surface disease, such as dry eye disease (DED) caused by reduced tear volume and/or increased tear evaporation. DED is characterized by dryness, burning, redness, excessive tearing, blurry vision, light sensitivity, and overall discomfort of the eye. Consequently, recent large questionnaire-based studies from diverse regions of the world indicate that DED is associated with worsened general health-related QoL (HRQoL) and that QoL scores worsen with more severe forms of the disease [2–9]. The prevalence of DED varies from 5 to 50%, depending on geographic region, study population, as well as definitions and diagnostic criteria employed [10]. DED is more common in women and older individuals and is associated with, e.g., certain autoimmune diseases, depression, and anxiety [10–13]. The economic burden of DED is also significant, mostly due to loss of work productivity [14].

Ocular surface health, including the presence of DED, can be evaluated by clinical examination and self-reported outcome questionnaires. Traditional clinical tests for DED include slit lamp examination, different ocular surface staining methods to detect defects on the corneal or conjunctival epithelium (Fluorescein, Lissamine green, and Rose Bengal), and tear film examinations to test tear stability and production (tear break-up time [TBUT], Schirmer test). It is also recommended to evaluate lid margin and meibomian gland health and test tear osmolarity. Multiple questionnaires have been developed for assessing the subjective symptoms of DED [14–16], the most prevalent being the Ocular Surface Disease Index (OSDI) questionnaire. Due to the multifactorial nature of DED, harmonized diagnostic criteria for DED are still missing; different continentals have different preferences, and the availability of diagnostic tools, and practices varies. These inconsistencies may hinder data pooling and meta-analyses, affecting the cohesive understanding of DED’s impact on QoL and the development of best practices. Recognizing QoL impacts in DED patients enables clinicians to tailor treatments to individual needs. For example, patients with work-related eye strain might benefit more from short breaks, and environment modifications, while those with significant inflammation may need anti-inflammatory medication. This patient-centered approach enhances treatment adherence and overall QoL.

Recently, we performed the first analysis of ocular surface health in Finland and showed that in elderly people, the prevalence of DED ranged from 10 to 33% based on the diagnostic criteria employed [17]. Furthermore, we observed distinct disparities in signs and symptoms of DED between men and women. The objective of the present study was to assess the association between QoL and ocular surface health in the same Finnish study cohort using both self-reported symptoms and clinical signs of DED and accounting for varying diagnostic criteria for DED. We hypothesized that sex differences influence the relationship between DED and QoL. This is the first population-based study combining general HRQoL data with objective clinical measures of DED.

## Methods

### Study population and design

**Fig. 1:**
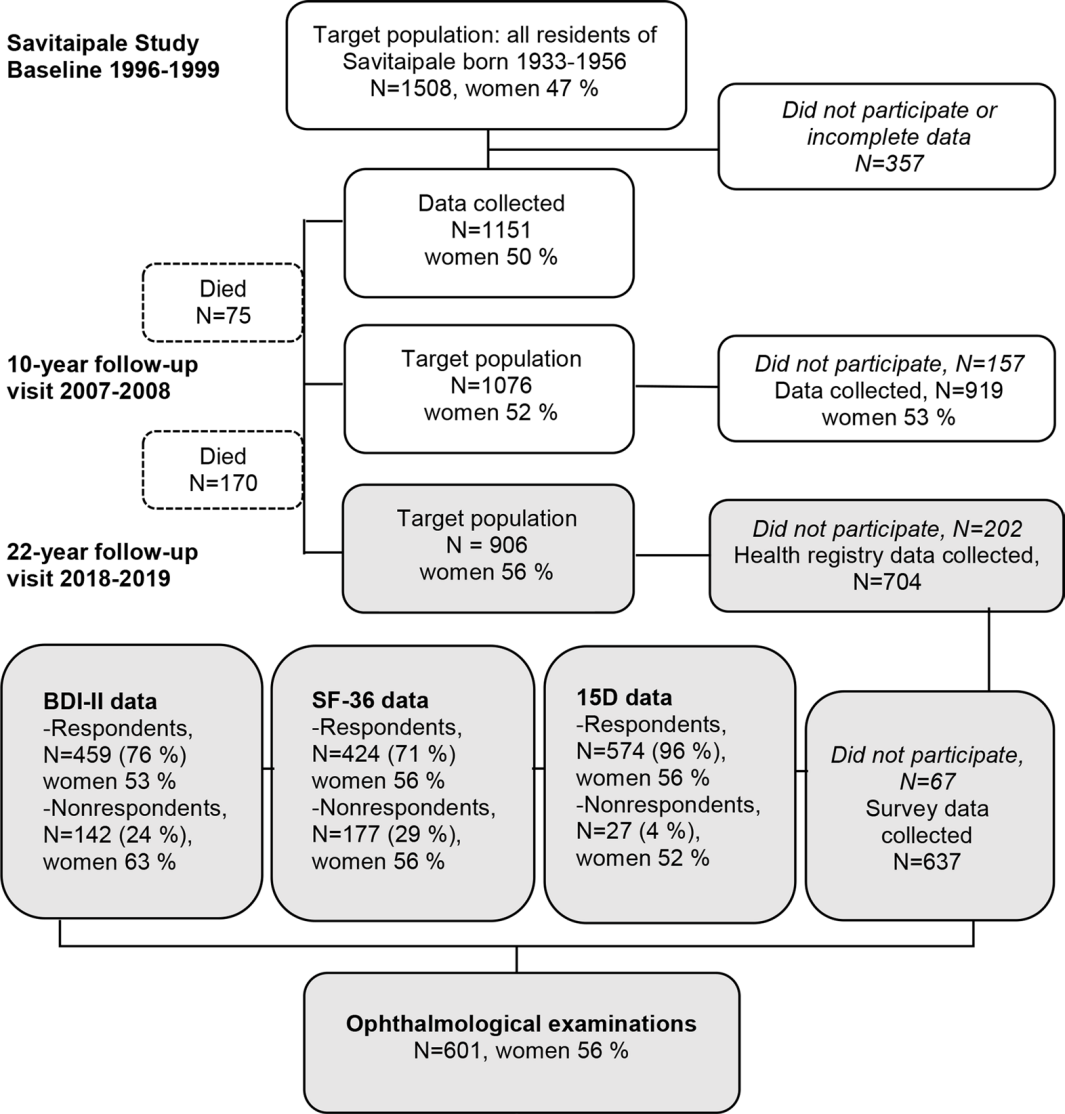
Sampling procedure of the study. Grey boxes indicate sampling during the 22-year follow-up visit when ophthalmological examinations and quality of life questionnaires (15D, SF-36 and BDI-II) were performed. Out of the 704 participants in the 22-year follow-up, 637 completed the survey. 67 consented only to the health registry data linkage due to poor health or reluctance. The percentages in the QoL data boxes are based on data from 601 participants who underwent ophthalmological examinations

A flowchart of the sampling procedure of the Savitaipale study is presented in Fig. 1. The details of the study can be found in previous publications [17–20]. The baseline visit was conducted in 1996–1999 when all individuals born between 1933 and 1956 and living in Savitaipale, Finland, on May 27, 1996, were invited to participate in the study. The follow-up visits were carried out 10 and 22 years after the baseline visit. Our cross-sectional population study included 601 participants (women = 335, men = 266) from the 22-year follow-up visit (Fig. 1). Participants filled in the QoL questionnaires during their visit with the study nurses and an ophthalmologist appointment was scheduled within two weeks. This study was conducted following the Declaration of Helsinki and was approved by the Research Ethics Committee of the Helsinki University Hospital (HUS/2203/2018). Written informed consent was obtained from all participants involved in the study. Previously, a cross-sectional ocular surface health study of this population at the 22-year follow-up visit has been reported [17]. Reporting results follow the Strengthening the Reporting of Observational Studies in Epidemiology (STROBE) guidelines [21].

### Measures

A comprehensive ophthalmological examination was performed, and ocular surface health was clinically evaluated using an extensive set of diagnostic tests. One ophthalmologist (IS) performed all examinations. A detailed description of the tests performed has been reported earlier [17]. In brief, best corrected visual acuity (BCVA), Schirmer’s I test, non-invasive tear break-up time (NIBUT), corneal (fluorescein) and conjunctival (Lissamine green) staining, conjunctival redness, blepharitis, and Meibomian gland dysfunction (MGD) were tested. Conjunctival redness was graded by using conjunctival redness reference photographs (scale 0–4), blepharitis and MGD by using the Efron scale (0–4), and corneal and conjunctival staining by using the Oxford grading (0–5). Thresholds for clinical signs in DED diagnosis were Schirmer’s test < 10s, NIBUT < 10s, corneal or conjunctival staining ≥ 1, conjunctival redness ≥ 2, blepharitis ≥ 2, and MGD ≥ 2. Dry eye symptoms were assessed using the Ocular Surface Disease Index (OSDI) questionnaire (0–100 scale), developed by the Outcomes Research Group at Allergan Inc in 1997 (Irvin, CA, USA). OSDI includes 12 items assessing vision-related function, ocular symptoms, and environmental triggers. All participants having OSDI score > 13, were considered to have DED symptoms. In addition, self-reported data on used medications and subjective dryness of the eyes (visual analog scale 0–10) were collected. The register-based data on common diseases (i.e., comorbidities) were obtained from the Finnish Care Register for Health Care.

### QoL questionnaires

For generic HRQoL measurements, the 15D and Medical Outcomes Study 36-Item Short Form Health Survey (SF-36) tools were used. Beck’s Depression Inventory (BDI-II) questionnaire was used to study the severity of depression.

The 15D is a generic HRQoL questionnaire with 15 dimensions/questions profiling mobility, vision, hearing, breathing, sleeping, eating, speech, elimination, usual activities, mental function, discomfort and symptoms, depression, distress, vitality, and sexual activity [22]. Each of 15 dimensions has a multiple choice (1–5) single-answer question contributing to a single index score (0–1 scale), with higher value representing better overall QoL. Missing data was imputed in R software as instructed by the developer of the 15D questionnaire and www.15D-instrument.net. The imputed data were considered to be complete (*N* = 574, 96%).

The SF-36 was developed at RAND as part of the Medical Outcomes Study [23]. The SF-36 measures the overall health and includes 36 questions divided into eight different categories: physical functioning, role-physical (i.e. problems with activities due to physical health), bodily pain, general health, vitality, social functioning, role-emotional (i.e. problems with activities due to emotional health), and mental health. In SF-36, a higher score (0–100 scale/question) represents better health status. The Physical Component Summary (PCS) and Mental Component Summary (MCS) were calculated for the SF-36 using questionnaire data without any missing items (*N* = 424, 71%) [24].

The BDI-II is a 21-item questionnaire measuring the severity of depression in adolescents and adults [25]. Each item corresponding to a symptom of depression has four answer options (0–3) which are summed to give a single score. In BDI-II, a higher score (0–63 scale) indicates more severe depression. BDI-II score was summed using questionnaire data without any missing items (*N* = 459, 76%).

### Dry eye diagnosis

Three different dry eye diagnostic criteria were used: OSDI, DE (dry eye), and OSD (ocular surface disease). The threshold for positive symptom-based dry eye diagnosis was OSDI score > 13. Diagnostic criteria for DE according to slightly modified guidelines of The Tear Film & Ocular Surface Society Dry Eye Workshop II report [26] included an OSDI score > 13 and at least one sign (NIBUT < 10 or corneal or conjunctival staining ≥ 1). OSD was diagnosed if at least two signs or symptoms were present (NIBUT < 10, corneal or conjunctival staining ≥ 1, Schirmer’s test < 10, OSDI > 13).

### Statistics

**Fig. 2:**
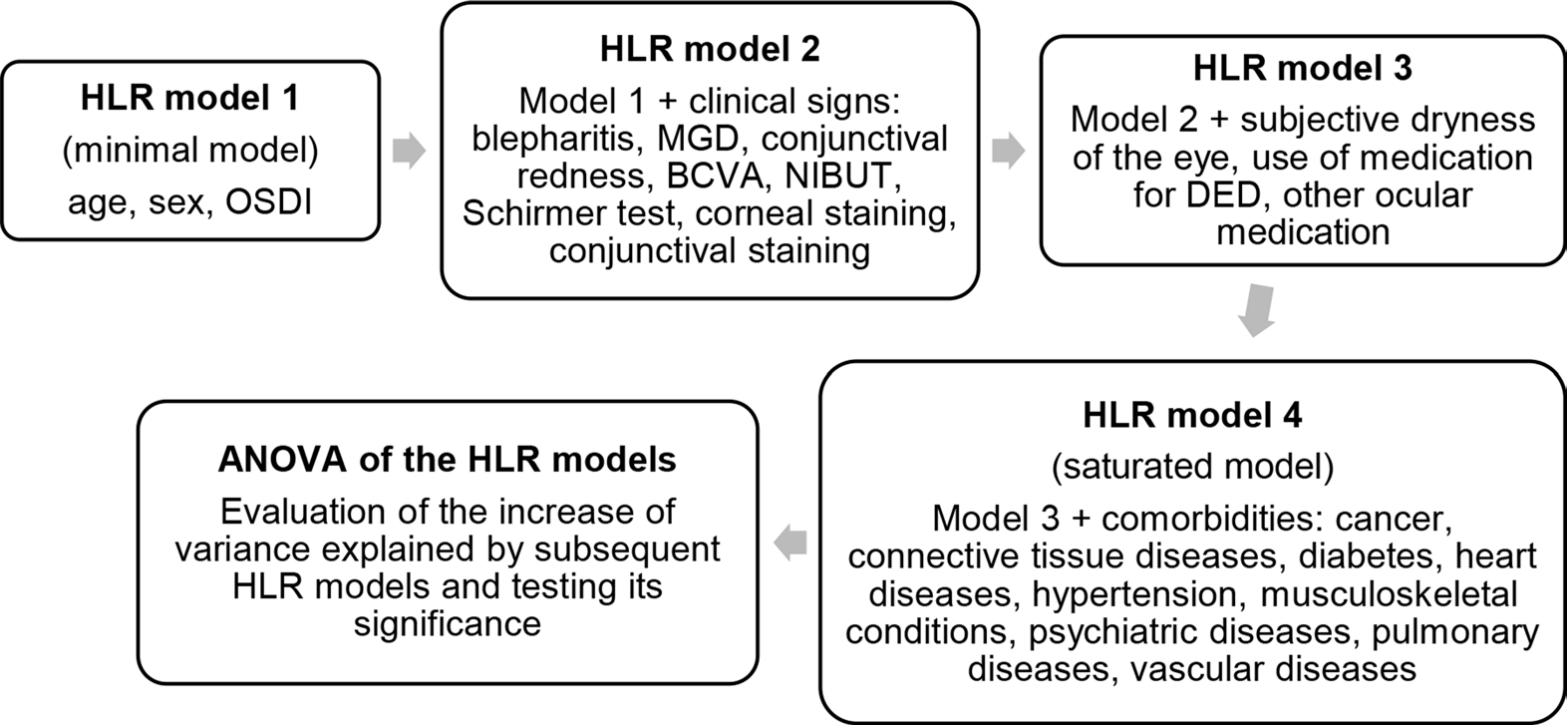
Hierarchical linear regression (HLR) analysis workflow, applied to identify factors with the greatest impact on quality of life scores. OSDI = ocular surface disease index, MGD = meibomian gland disease, BCVA = best corrected visual acuity, NIBUT = noninvasive tear break-up time, DED = dry eye disease

For statistical analyses of the ocular surface health parameters, the worst eye based on Schirmer’s I test, NIBUT, and corneal staining was selected. The best corrected visual acuity (BCVA) from the eye with better vision was used for statistical analyses. The reliability of the QoL data was investigated through floor and ceiling effects, and internal consistency. The internal consistency of 15D and BDI-II was assessed using Cronbach’s alpha, with a threshold of 0.7 or higher considered acceptable, and internal consistency coefficients of SF36 PCS and MCS were estimated using the instructions provided by Ware et al., 1993 [24]. The internal validity of QoL scores as well as the association between the ocular surface signs, and QoL scores were investigated with correlation analysis. Spearman correlation was used for ordinal variables, and Pearson’s correlation was used for continuous variables. Age-differences, BCVA, DED symptoms and QoL scores between sexes were evaluated using the Wilcoxon rank sum test for non-normal data (normality tested with the Shapiro-Wilk test). The difference in QoL scores between people with positive and negative DED diagnosis using three different DED diagnostic criteria (DE, OSD, OSDI) was investigated using the Wilcoxon rank sum test. We applied multiple logistic regression to find the odds ratios (OR) and adjusted OR of having worse QoL due to DED, using three different diagnostic criteria (OSDI, DE, OSD) for DED. For OR analysis, we dichotomized QoL scores based on median: SF36 PCS, SF36 MCS and 15D below the median, and BDI-II above median were considered worsened QoL. The OR analysis was performed to facilitate comparisons with findings from previous publications. To mitigate the loss of information due to dichotomization, we repeated the OR analysis by stratifying QoL data based on quartiles. Hierarchical linear regression (HLR) was used to further examine the independent effects of ocular surface health on QoL (Fig. 2). We chose HLR because we wanted to investigate the incremental contribution of levels of factors - demographic and OSDI, clinical variables, DED medication use, and comorbidities - to the explained variance in the quality of life (QoL), while controlling for confounding factors and potential dependencies within each level. All analyses were performed with R (version 4.3.1).

### Comorbidities

For OR analysis and HLR, we included common diseases as confounders to account for their possible impact on HRQoL. The list of inpatient and outpatient ICD-10 (International Classification of Diseases 10th Revision) diagnoses were obtained from the Finnish Care Register for Health Care and classified into 9 main comorbidity categories: “Cancer”, “Connective tissue diseases”, “Diabetes”, “Heart diseases”, “Hypertension”, “Musculoskeletal conditions”, “Psychiatric diseases”, “Pulmonary diseases”, and “Vascular diseases”. ICD-10 codes, which did not match any of these main categories, were excluded from the analysis. A comorbidity was considered diagnosed in a study participant if at least one of the ICD-10 codes falling within the main category was found to be diagnosed in the participant at any time point. The ICD-10 codes and the prevalence of each comorbidity group are presented in Online Resource 1.

### Missing data and sensitivity analysis

We performed a sensitivity analysis to assess the potential impact of missing QoL data on our results. The nature of missing data and sensitivity analysis workflow are described in Online Resource 2.

## Results

Ophthalmological examination results were obtained from > 99% of the 601 participants, with only single values missing from different tests. The QoL mean scores, BCVA, and subjective ocular surface health measures of the study population are described in Table 1. QoL mean scores revealed significant sex differences in mental health (SF-36 MCS and BDI-II). In addition to SF-36 MCS and BDI-II scores, SF-36 dimensions measuring energy/fatigue, emotional well-being, social functioning, and pain, indicated better QoL scores in men than women. Men had slightly better vision (BCVA) and experienced fewer DED symptoms (subjective eye dryness and OSDI) compared with women. Detailed results of the ophthalmologic examination in this study population and the data on the use of the medication for DED have been published previously [17].

**Table 1:** Means of quality of life scores and ocular surface health measures in all participants and by sex

Measure	Unit of measure (range)	All, mean (SD)	Women, mean (SD)	Men, mean (SD)	Difference, women vs. men, *p*-value^1^
N		601	335	266	
Age	year (62–86)	72.0 (6.30)	72.3 (6.33)	71.6 (6.25)	0.120
BCVA	Snellen chart (0–1)	0.88 (0.16)	**0.86 (0.16)**	**0.89 (0.15)**	**0.026**
Subjective eye dryness	cm (0–10)	3.4 (2.8)	**3.9 (2.8)**	**2.6 (2.7)**	**< 0.001**
OSDI	OSDI score (0–100)	8.6 (9.6)	**10.4 (11.0)**	**6.38 (7.0)**	**< 0.001**
15D N	0–1	0.90 (0.09) 574	0.89 (0.09) 321	0.90 (0.09) 253	0.212
SF-36 Dimension N	0-100 Physical functioning	76.27 (24.82) 489	75.11 (24.63) 274	77.75 (25.04) 215	0.093
	Role physical	68.70 (41.00) 483	68.33 (41.26) 270	69.17 (40.76) 213	0.924
	Role emotional	79.59 (36.07) 485	79.04 (36.50) 272	80.28 (35.57) 213	0.721
	Energy/fatigue	**70.31 (36.07)** 483	**68.19 (19.13)** 265	**72.87 (21.74)** 218	**0.002**
	Emotional well-being	**80.36 (16.96)** 461	**78.76 (16.48)** 255	**82.34 (17.37)** 206	**0.001**
	Social functioning	**86.71 (19.44)** 551	**84.76 (21.06)** 310	**89.21 (16.86)** 241	**0.025**
	Pain	**79.74 (19.21)** 559	**77.85 (20.52)** 312	**82.12 (17.15)** 247	**0.034**
	General health	57.91 (20.66) 558	57.03 (20.64) 310	59.01 (20.67) 248	0.495
SF-36 PCS SF-36 MCS N	0-100	46.11 (9.47) 53.91 (9.49) 424	45.84 (9.98) **53.02 (9.76)** 236	46.45 (8.80) **55.02 (9.03)** 188	0.758 **0.011**
BDI-II N	0–63	5.78 (0.61) 459	**6.32 (5.19)** 245	**5.16 (5.41)** 214	**0.001**

^1^ Wilcoxon rank sum test. SD = standard deviation, N = number of participants, BCVA = best corrected visual acuity, OSDI = ocular surface disease index, PCS = physical component summary, MCS = mental component summary, BDI = Beck’s depression inventory

### Quality of life questionnaire data

Before imputation response rates were 91–94% for each 15D item, except for the sexual activity item (79%). After imputing missing items, the number of participants eligible for analysis was 574 (96%) for the 15D. Full responses were obtained from 424 participants (71%) for the SF-36 questionnaire and 459 participants (76%) for the BDI-II (Fig. 1; Table 1). The potential impact of missing QoL data was assessed by examining the pattern of missing data, comparing the outcomes between responders and non-responders, and conducting sensitivity analysis using best-case and worst-case scenarios. The results on the potential impact of missing data are available in Online Resource 2. Generally, the non-responders tended to be older than the responders, which was acknowledged through age adjustments in further analyses. The reliability and validity estimate for QoL scores are presented in Online Resource 3. Internal consistencies of the 15D (0.82), SF-36 PCS (0.92), SF-36 MCS (0.88) and BDI-II (0.85) scores were excellent or good.

### The association between ocular surface signs and symptoms with quality of life

The correlation of ocular surface signs and symptoms of DED with QoL is presented in Table 2. OSDI was associated with each QoL score in all study participants; a higher OSDI score correlated with a worse QoL. In women, OSDI correlated with all QoL scores except the SF-36 MCS, whereas in men, the OSDI only correlated with the 15D. Among all participants, the most significant ocular surface sign correlating with all four QoL mean scores (15D, SF-36 PCS, SF-36 MCS, and BDI-II) was NIBUT; shorter values correlated with worse QoL score. Short NIBUT indicated that tear film on the ocular surface was breaking up too quickly causing issues with tear quality or distribution and thereby e.g. blurred vision, stinging sensation in eyes, watery eyes or photophobia for study participants. Higher conjunctival staining grades correlated with worse 15D and BDI-II scores, mainly in women. High conjunctival staining grade indicated the damage in epithelial cells of the conjunctiva, a thin layer of tissue covering the anterior part of the sclera and the inner surface of eyelids, which could cause irritation, redness and discomfort in the eyes of participants. Additionally, in women, an association was observed between the short NIBUT and worse 15D and SF-36 MCS scores. Among men, blepharitis correlated with the depression score. Blepharitis indicated inflammation of the eyelids causing red, itchy eyelids and formation of dandruff-like scales on the eyelashes in men. According to the 15D, a good BCVA correlated with a high HRQoL in general.

**Table 2:** The correlation between the ocular surface signs and symptoms and the quality of life scores in all study participants (all), in women (W) and men (M)

		Correlation coefficient^1^ Adjusted *P*– value								
		OSDI	Blepharitis	MGD	Conjunctival redness	Corneal staining	Conjunctival staining	NIBUT	Schirmer test	BCVA
15D, *N* = 574	All	**-0.25**	-0.05	-0.01	-0.06	**-0.10**	**-0.15**	**0.15**	-0.05	**0.22**
		**< 0.001**	0.252	0.885	0.225	**0.044**	**0.002**	**0.001**	0.252	**< 0.001**
	W	**-0.28**	-0.06	0.03	-0.04	-0.10	**-0.19**	**0.20**	-0.01	**0.21**
		**< 0.001**	0.365	0.666	0.615	0.154	**0.004**	**0.002**	0.847	**0.001**
	M	**-0.20**	-0.07	-0.07	-0.11	-0.09	-0.08	0.08	-0.11	**0.23**
		**0.035**	0.378	0.339	0.171	0.285	0.300	0.339	0.171	**0.010**
SF-36 PCS, *N* = 424	All	**-0.17**	-0.01	0.09	-0.10	0.03	-0.10	**0.13**	-0.09	0.10
		**0.002**	0.856	0.110	0.070	0.684	0.070	**0.024**	0.110	0.092
	W	**-0.21**	0.08	0.11	-0.04	0.09	-0.09	0.11	-0.03	0.07
		**0.005**	0.302	0.175	0.665	0.282	0.285	0.175	0.754	0.419
	M	-0.07	-0.14	0.04	-0.19	-0.07	-0.12	0.15	-0.17	0.14
		0.429	0.155	0.608	0.080	0.429	0.171	0.137	0.102	0.155
SF-36 MCS, *N* = 424	All	**-0.15**	-0.09	-0.02	-0.10	-0.06	-0.10	**0.14**	-0.02	0.03
		**0.006**	0.094	0.750	0.092	0.252	0.070	**0.010**	0.750	0.577
	W	-0.14	-0.11	0.08	-0.10	-0.07	-0.12	**0.18**	0.03	0.01
		0.086	0.175	0.345	0.255	0.419	0.175	**0.026**	0.735	0.917
	M	-0.12	-0.13	-0.18	-0.17	-0.02	-0.08	0.10	-0.07	0.05
		0.171	0.171	0.102	0.102	0.840	0.382	0.300	0.427	0.558
BDI-II, *N* = 459	All	**0.19**	0.08	0.02	0.08	0.09	**0.18**	**-0.12**	0.02	**-0.13**
		**< 0.001**	0.110	0.750	0.128	0.095	**0.001**	**0.031**	0.750	**0.017**
	W	**0.20**	0.02	-0.05	0.08	0.10	**0.27**	-0.14	-0.01	-0.12
		**0.008**	0.758	0.615	0.302	0.239	**< 0.001**	0.086	0.847	0.138
	M	0.13	**0.20**	0.13	0.15	0.05	0.06	-0.09	0.03	-0.11
		0.155	**0.036**	0.155	0.124	0.558	0.429	0.300	0.659	0.177

^1^Correlation coefficients were calculated for each individual variable using Spearman correlation for ordinal variables and Pearson correlation for continuous variables. OSDI = ocular surface disease index; MGD = meibomian gland disease, NIBUT = noninvasive tear film break-up time, BCVA = best corrected visual acuity, N = number of participants, PCS = physical component summary, MCS = mental component summary, BDI = Beck’s depression inventory

### Dry eye diagnosis and quality of life

To assess the impact of DED and its different diagnostic criteria on the QoL mean scores, we divided the study population into DED positive and negative groups using OSDI, DE, and OSD diagnostic criteria (Table 3). DED had a significant negative impact on each QoL score in the complete study population regardless of the diagnostic criteria applied, except on the SF-36 PCS score when DE criteria were applied. Across all diagnostic categories, DED was more common in women, and they reported lower QoL scores compared with men. Among men, only OSDI diagnostic criteria resulted in significant differences in 15D and BDI-II scores, while the effect on SF-36 scores was not significant. The other DED diagnostic categories (DE or OSD) were not associated with the QoL measures in men. Overall, the symptom-based OSDI diagnosis category resulted in the largest differences in all QoL measures in both sexes, whereas in the DE diagnosis category, considering both symptoms and signs, only a few significant differences were observed between people with and without DED.

**Table 3:** The quality of life (QoL) mean scores of dry eye positive and negative participants according to different diagnostic categories (OSDI, DE, OSD)

		All, mean score (SD)			Women, mean score (SD)			Men, mean score (SD)		
Diagnosis category	QoL ques- tionnaire	Pos^1^	Neg	*p*-val	Pos	Neg	*p*-val	Pos	Neg	*p*-val
OSDI^3^ Pos All 21% W 28% M 13%	15D	**0.86 (0.09)**	**0.91 (0.08)**	**< 0.001**	**0.86 (0.10)**	**0.91 (0.08)**	**< 0.001**	**0.87 (0.09)**	**0.90 (0.08)**	**0.028**
	SF-36 PCS	**44.0 (9.9)**	**46.7 (9.3)**	**0.021**	**43.0 (10.1)**	**47.0 (9.7)**	**0.006**	46.8 (8.7)	46.4 (8.85)	0.922
	SF-36 MCS	**51.3 (10.1)**	**54.6 (9.2)**	**< 0.001**	**50.9 (10.5)**	**53.9 (9.4)**	**0.023**	52.4 (9.2)	55.4 (9.0)	0.051
	BDI-II	**7.6 (5.9)**	**5.3 (5.1)**	**< 0.001**	**7.6 (5.9)**	**5.8 (4.8)**	**0.011**	**7.4 (6.2)**	**4.9 (5.2)**	**0.025**
DE^4^ Pos All 10% W 14% M 5%	15D	**0.86 (0.10)**	**0.90 (0.08)**	**< 0.001**	**0.85 (0.10)**	**0.90 (0.08)**	**< 0.001**	0.88 (0.10)	0.90 (0.09)	0.344
	SF-36 PCS	44.9 (9.8)	46.3 (9.4)	0.436	44.5 (10.1)	46.1 (10.0)	0.403	45.9 (9.5)	46.5 (8.8)	0.928
	SF-36 MCS	**51.9 (9.1)**	**54.1 (9.5)**	**0.029**	51.3 (10.0)	53.3 (9.7)	0.167	53.4 (6.5)	55.1 (9.2)	0.163
	BDI-II	**7.6 (6.1)**	**5.6 (5.2)**	**0.011**	7.9 (6.2)	6.1 (5.0)	0.052	6.5 (5.7)	5.1 (5.4)	0.307
OSD^5^ Pos All 33% W 37% M 28%	15D	**0.88 (0.09)**	**0.91 (0.08)**	**< 0.001**	**0.87 (0.09)**	**0.91 (0.08)**	**< 0.001**	0.89 (0.09)	0.90 (0.08)	0.288
	SF-36 PCS	**44.4 (10.4)**	**47.0 (8.8)**	**0.016**	**44.0 (10.9)**	**47.0 (9.2)**	**0.038**	45.1 (9.6)	47.0 (8.4)	0.202
	SF-36 MCS	**52.2 (10.3)**	**54.8 (8.9)**	**0.006**	51.6 (10.5)	53.9 (9.2)	0.078	53.0 (9.9)	55.8 (8.6)	0.065
	BDI-II	**6.8 (6.0)**	**5.3 (4.9)**	**0.022**	**7.3 (5.6)**	**5.8 (4.9)**	**0.021**	6.0 (6.5)	4.9 (4.9)	0.577

^1^Pos indicates positive dry eye diagnosis and Neg negative dry eye diagnosis, women (W) and men (M). Statistically significant results (Wilcoxon rank sum test, p-val < 0.05) are in bold. ^3^Positive = OSDI > 13; ^4^Positive = OSDI > 13 and at least one sign: NIBUT < 10, corneal or conjunctival staining > 0; ^5^Positive = at least two signs or symptoms: NIBUT < 10, corneal or conjunctival staining > 0, Schirmer test < 10 or OSDI > 13. Number of participants in each QoL questionnaire data analysis is presented in Table 1. SD = standard deviation, OSDI = ocular surface disease index, DE = dry eye, OSD = ocular surface disease, PCS = physical component summary, MCS = mental component summary, BDI = Beck’s depression inventory

Next, we verified whether DED remained as a risk factor for worsened QoL when adjusting for age, sex, BCVA, and comorbidities (Table 4). We used the OSDI, DE, and OSD diagnostic criteria and categorized the data into lower and higher QoL based on the mean. Compared with individuals without DED, the OR of a lower 15D score was higher among individuals with DED, regardless of the criteria chosen (adjusted OR 1.95–2.51). Similarly, the adjusted OR of worse mental health score was approximately two times higher among people with DED than those without across all definitions of DED according to the BDI-II score, and when the OSDI and DE criteria were applied according to SF-36 MCS. However, DED did not increase the odds of worse physical health (SF-36 PCS), when adjusted for comorbidities, sex, age, and BCVA. In general, when compared with men, women did not have significantly higher odds of experiencing lower QoL, except for BDI-II, where the adjusted OR of the increased depression score was 1.5 times higher in women (DE adjusted OR 1.5, 95% CI 1.00 to 2.24, p-value < 0.05, OSD adjusted OR 1.51, 95% CI 1.01 to 2.26, p-value < 0.05). To minimize possible information loss caused by dichotomizing QoL scores, we also conducted the OR analysis using QoL score quartiles (Online Resource 4). The ordinal logistic regression results were in keeping with the results from logistic regression presented in Table 4 and the conclusion, that DED imposed a significant risk of worse QoL.

**Table 4:** The adjusted odds ratios (OR) of the low general health-related quality of life (QoL) score or high depression score in people with dry eye disease (DED) in comparison with those without DED according to three different DED diagnostic criteria (OSDI, DE, OSD)

	OSDI OR (95% CI)	DE OR (95% CI)	OSD OR (95% CI)
15D, *N* = 574	**2.51 (1.58**,** 3.99)** ***	**2.31 (1.24**, 4.31) **	**1.95 (1.33**, 2.87) ***
SF-36 PCS, *N* = 424	1.44 (0.84, 2.46)	1.46 (0.71, 3.02)	1.5 (0.95, 2.36)
SF-36 MCS, *N* = 424	**1.95 (1.18**, 3.23) **	**2.08 (1.04**, 4.16) *	1.36 (0.9, 2.07)
BDI-II, *N* = 459	**1.88 (1.13**, 3.11) *	**2.08 (1.04**, 4.16) *	**1.59 (1.03**, 2.45) *

QoL scores were dichotomized based on the median score. OR was adjusted for age, sex, BCVA and comorbidities, 95% confidence intervals are shown in the parentheses, and statistical significance (Wald’s test) is denoted by asterisks: * p-value < 0.05, ** p-value < 0.01, *** p-value < 0.001. DED = dry eye disease, OSDI = ocular surface disease index, DE = dry eye, OSD = ocular surface disease, BCVA = best corrected visual acuity, CI = confidence interval, PCS = physical component summary, MCS = mental component summary, BDI = Beck’s depression inventory. DED diagnostic criteria can be found in Table 3

### Hierarchical linear regression analysis

Since the initial analyses indicated that OSDI and clinical signs of DED have an impact on QoL, we further investigated this association using HLR, to account for the possible impact of comorbidities and DED and other ocular medication use. In addition, due to observed differences between sexes in DED, we conducted analyses for men and women separately. We performed forward HLR and compared four sequential models (Models 1–4), starting from a minimal model including only age, sex, and OSDI, and ending with a saturated model (Fig. 2). The associations of all DED symptoms and signs with QoL scores from each model are shown in the table of Online Resource 5. The variance inflation factor (VIF) ranges of the saturated models were 1.06–1.79 for all participants, 1.07–1.89 for women, and 1.13–1.93 for men, which means negligible or very little multicollinearity. ANOVA analysis of the HLR models revealed that in most cases, explained variance increased significantly after the inclusion of new variables after at least one step of the process. However, expanding models predicting SF-36 MCS in women, and models predicting BDI-II in men did not improve the variance explained. As expected, we found that increased OSDI remained significant in nearly all models, especially in women as well as in all participants, exerting a negative impact on all QoL scores (Table 5). Among men, OSDI was significantly associated only with 15D and BDI-II scores, but the associations were not significant when adjusted for comorbidities. Subjective dryness of the eyes negatively affected SF-36 MCS among all participants and in men, as well as 15D in women. Among clinical signs, increased conjunctival staining worsened BDI-II score among women, whereas MGD was associated with a lower SF-36 MCS among men (Table 6). Age was associated with most scores but was the least important variable for the SF-36 summary scores among men and SF-36 MCS among women (data not shown).

**Table 5:** Impact of ocular surface disease index (OSDI) on quality of life scores (15D, SF-36 PCS, SF-36 MCS, BDI-II) in hierarchical linear regression models 1–4^1^

ALL					
		Model 1	Model 2	Model 3	Model 4
**15D** *N* = 574	B coef.^2^	**-0.002**	**-0.002**	**-0.002**	**-0.002**
	Beta coef.	**-0.260**	**-0.253**	**-0.204**	**-0.194**
	Adj. p-value	< 0.001***	< 0.001***	< 0.001***	< 0.001***
**SF-36 PCS** *N* = 424	B coef.	**-0.165**	**-0.175**	**-0.149**	**-0.123**
	Beta coef.	**-0.167**	**-0.177**	**-0.151**	**-0.125**
	Adj. p-value	0.001**	0.002**	0.024*	0.044*
**SF-36 MCS** *N* = 424	B coef.	**-0.134**	**-0.153**	**-0.137**	-0.149
	Beta coef.	**-0.135**	**-0.154**	**-0.139**	-0.150
	Adj. p-value	0.009**	0.026*	0.041*	0.056
**BDI-II** *N* = 459	B coef.	**0.097**	**0.100**	**0.092**	**0.095**
	Beta coef.	**0.170**	**0.174**	**0.160**	**0.165**
	Adj. p-value	< 0.001***	0.001**	0.008**	0.008**
**WOMEN**					
		Model 1	Model 2	Model 3	Model 4
**15D** *N* = 321	B coef.	**-0.002**	**-0.002**	**-0.002**	**-0.002**
	Beta coef.	**-0.284**	**-0.276**	**-0.207**	**-0.232**
	Adj. p-value	< 0.001***	< 0.001***	0.002**	< 0.001***
**SF-36 PCS** *N* = 236	B coef.	**-0.184**	**-0.213**	**-0.167**	**-0.188**
	Beta coef.	**-0.203**	**-0.235**	**-0.184**	**-0.208**
	Adj. p-value	0.001**	0.002**	0.042*	0.021*
**SF-36 MCS** *N* = 236	B coef.	**-0.122**	-0.156	-0.146	-0.147
	Beta coef.	**-0.138**	-0.176	-0.165	-0.166
	Adj. p-value	0.032*	0.111	0.229	0.313
**BDI-II** *N* = 245	B coef.	**0.092**	**0.091**	0.077	0.085
	Beta coef.	**0.188**	**0.186**	0.157	0.175
	Adj. p-value	0.002**	0.012*	0.085	0.107
**MEN**					
		Model 1	Model 2	Model 3	Model 4
**15D** *N* = 253	B coef.	**-0.003**	**-0.003**	**-0.002**	-0.002
	Beta coef.	**-0.210**	**-0.199**	**-0.180**	-0.140
	Adj. p-value	< 0.001***	0.006**	0.031*	0.280
**SF-36 PCS** *N* = 188	B coef.	-0.097	-0.054	-0.059	0.052
	Beta coef.	-0.075	-0.041	-0.046	0.040
	Adj. p-value	0.292	0.743	0.788	0.768
**SF-36 MCS** *N* = 188	B coef.	-0.169	-0.172	-0.147	-0.138
	Beta coef.	-0.127	-0.129	-0.110	-0.103
	Adj. p-value	0.094	0.397	0.347	0.484
**BDI-II** *N* = 214	B coef.	**0.112**	0.109	0.107	0.097
	Beta coef.	**0.140**	0.136	0.134	0.121
	Adj. p-value	0.038*	0.195	0.185	0.327

^1^Model 1 is adjusted for age, sex, and OSDI. Model 2 includes covariates in model 1 plus clinical signs. Model 3 includes covariates in model 2 plus subjective dryness of the eyes, use of medications for DED and other ocular medication. Model 4 includes covariates in model 3 and comorbidities listed in Online Resource 1. ^2^The B coefficients are unstandardized showing the magnitude of the impact on QoL. The Beta coefficients are standardized and can be compared with each other. P-values were adjusted using Benjamini-Hochberg correction method. Statistically significant p-values are bold, and the significance level is marked with asterisks: *p-value < 0.05, **p-value < 0.01, ***p-value < 0.001. PCS = physical component summary, MCS = mental component summary, BDI = Beck’s depression inventory

**Table 6:** Impact of dry eye disease symptoms and signs on Beck’s depression inventory (BDI-II) score in women and SF-36 mental component summary (MCS) in men using hierarchical linear regression models 1–4 (M1-4)^1^

	Women, BDI-II, *N* = 245				Men, SF-36 MCS, *N* = 188			
	M1	M2	M3	M4	M1	M2	M3	M4
Blepharitis		-0.081	-0.089	-0.060		-0.012	0.011	-0.012
MGD		0.054	0.051	0.055		-0.221	**-0.238**	-0.209
Conjunctival redness		0.024	0.026	0.054		-0.061	0.105	0.066
BCVA		0.046	0.041	0.021		-0.067	-0.046	-0.036
NIBUT		-0.001	-0.006	0.001		0.038	0.025	0.006
Schirmer		0.014	0.010	-0.049		-0.064	-0.057	-0.014
Corneal staining		0.071	0.066	0.088		-0.027	-0.035	-0.010
Conjunctival staining		**0.217**	**0.209**	0.166		-0.008	0.006	0.015
OSDI	**0.188**	**0.186**	0.157	0.175	-0.127	-0.129	-0.110	-0.103
Subjective eye dryness			0.145	0.120			**-0.272**	**-0.282**
DED med. freq.			-0.052	-0.031			-0.203	0.193
Other ocular med. use			-0.051	-0.092			0.020	0.053
Cancer				0.112				-0.095
Connective tissue diseases				0.038				-0.031
Diabetes				0.091				-0.111
Heart diseases				0.111				0.135
Hypertension				0.050				-0.008
Musculoskeletal conditions				-0.066				0.077
Psychiatric diseases				0.131				-0.120
Pulmonary diseases				-0.049				-0.073
Vascular diseases				-0.145				-0.043
R^2^	**0.11**	**0.17**	**0.19**	**0.26**	**0.06**	**0.11**	**0.14**	**0.20**
R^2^ change		**0.06**	0.02	**0.07**		0.05	0.03	0.06
Adjusted R^2^	**0.10**	**0.14**	**0.14**	**0.18**	**0.05**	**0.07**	**0.08**	**0.10**
Adjusted R^2^ change		**0.04**	0.01	**0.04**		0.01	0.02	0.02

^1^The table presents standardized Beta coefficients from hierarchical linear regression. Bold coefficients are statistically significant (adjusted p-value < 0.05). P-values were adjusted using Benjamini-Hochberg correction method. The table presents also R^2^ and adjusted R^2^ values and their change between successive models. Bold R^2^ and adjusted R^2^ values mean that the overall p-value was significant (< 0.05). Bold changes were statistically significant according to ANOVA analysis of the models (p-value < 0.05). Model 1 is adjusted for age, sex, and OSDI. Model 2 includes covariates in model 1 plus clinical signs of DED. Model 3 includes covariates in model 2 plus subjective dryness of the eyes, use of medications for DED and other ocular medication. Model 4 includes covariates in model 3 and comorbidities (a full list of comorbidities in Online resource 1). BCVA = best corrected visual acuity, NIBUT = noninvasive tear break-up time, OSDI = ocular surface disease index, DED med. freq.=dry eye disease medication frequency

## Discussion

Large population-based studies on DED often depend on data reported by the participants [2–9]. The uniqueness of our population study lies in its comprehensive methodology, i.e., both subjective and objective measures of DED were compared with HRQoL data obtained from two different generic questionnaires and a separate depression questionnaire. We also used three different diagnostic criteria for DED in the QoL analyses; OSDI diagnostic criteria were based on self-reported DED symptoms, whereas OSD diagnosis considered symptoms or clinical signs, and DE required both symptoms and signs for positive DED diagnosis. Consistent with previously published findings, women exhibited a higher prevalence of DED compared with men and this trend was observed across all diagnostic categories. Regardless of the diagnostic criteria used DED was consistently associated with reduced mean QoL scores, although the findings displayed some degree of variability. Overall, OSDI, the symptom-based diagnosis generated the largest decrease in QoL mean scores among individuals with or without DED. OSD, and DE diagnoses indicated worsening of QoL among women but not in men. The different diagnosis criteria for DED vary in their sensitivity and specificity, leading to differences in classification of individuals and, also impacting the QoL data interpretation. Diagnostic criteria also guide treatment, so inconsistent thresholds may result in disparities in the management of DED. Hence, the effects of different diagnosis criteria for DED and sex should be acknowledged when comparing findings from studies on DED-related QoL.

Sex and sex hormones play a significant role in DED, as emphasized in comprehensive reviews by Sullivan [27] and Matossian [28]. In addition to DED symptoms, more attention has recently been paid to sex-specific clinical signs among people with DED [29–32]. There is some disparity in findings, but overall, in DED studies women tended to have higher corneal staining [29, 32], shorter tear break-up time (TBUT) [29, 30, 32], and increased tear osmolarity [29, 30], while men experienced more conjunctival redness [31]. The first stages of our analysis revealed that several individual DED symptoms and signs correlated with QoL scores. In all participants, higher OSDI and corneal and conjunctival staining grades as well as short NIBUT were associated with worse QoL scores. At the population level, increased DED symptoms have been associated with worse general health- and vision-related QoL [9–18, 33, 34], but very little data are available on how clinical signs may affect these outcomes. A population-based cross-sectional study of 229 participants [33] concluded that none of the clinical variables of DED correlated with the vision-related QoL score. Similarly, two HRQoL studies concentrating only on DED patients (less than 160 individuals), did not find a correlation with clinical signs [35, 36]. We identified sex-differences in ocular surface signs affecting QoL scores. In women, high OSDI, conjunctival staining, and short NIBUT correlated with worse QoL scores. Among men, increased blepharitis correlated with higher depression scores. These QoL-associated symptoms and signs of DED were somewhat similar to our initial findings [17] indicating that women of this study population had higher OSDI, and corneal staining scores compared with men, while men more frequently showed signs of meibomian gland disease (MGD), blepharitis and conjunctival redness. In addition to recognizing sex differences in these associations, it is evident that a sufficiently large study population is necessary to observe the impact of clinical ocular variables on QoL measures. DED is a complex disease and understanding sex differences in DED can aid in selecting treatment options and thereby improving the QoL of patients. On basis of our previous and current results, for men in this study population, the priority for better ocular surface health-related QoL might be initiating and encouraging using the tear substitutes and taking care of lid hygiene, while women could benefit more from gaining insight into the pathogenesis and management of DED symptoms.

When comorbidities of all study participants were controlled for in HLR analysis, subjective symptoms (OSDI) still significantly affected QoL (15D, SF-36 PCS, and BDI-II) scores. Sex-specific analyses indicated that in women, OSDI was associated with 15D and SF-36 PCS, while among men, subjective eye dryness was associated with SF-36 MCS. Increased conjunctival staining score was associated with worse BDI-II among women, whereas MGD was associated with lower SF-36 MCS among men. By including comorbidities in regression analysis, we observed a significantly reduced association between DED and QoL, which aligns with the understanding that DED can be linked to various systemic diseases and medications used for their treatment [37–41]. The statistical approaches used in the analysis are sensitive to changes in the sample size, which can also play a role in the reduced associations observed. Furthermore, it is well-established that clinical findings and subjective symptoms in DED show poor correlation [42]. We found a significant association of DED symptoms with QoL, but inconsistent results with clinical signs. This suggests a similar paradigm than observed with DED symptoms and signs: clinical signs poorly predict the subjective wellbeing of people with DED.

In a large population-based study (*n* = 78 165) performed with self-reported data in the Netherlands, the OR of worse QoL in people with DED was 1.54 according to SF-36 PCS and 1.39 according to SF-36 MCS when comorbidities were not included in the statistical analysis [5]. The odds decreased to 1.22 after adjusting for comorbidities. Regardless of diagnostic criteria used, in our study population, DED diagnosis was associated with approximately two-fold higher odds of worse QoL after adjusting for comorbidities when compared with people without DED. This indicates that DED itself has a serious impact on the QoL of older individuals, whether detected by subjective symptoms or objective clinical signs. The results also imply that both symptoms and clinical signs of DED impact QoL. The Beaver Dam Offspring Study in the USA [3] and the DED prevalence survey in Germany [7] found lower physical and mental SF-36 scores in people with DED than in controls. Contrary to these population-based studies, in our study DED neither influenced the SF-36 PCS nor increased the odds of worse physical health (SF-36 PCS). The study populations in the Netherlands, USA, and Germany comprised on average of younger participants and the number of participants was higher than in our study, but DED was only diagnosed through a self-administered questionnaire. These factors may have contributed to the differences in QoL outcomes among different study populations.

Several systematic reviews [12, 13, 43] have revealed a significant correlation between DED symptoms, depression, and anxiety. An association was found to be bidirectional, suggesting that depression can both contribute to and result from DED [4]. Wan et al. [43] indicated that the prevalence of depression and anxiety could be even three times higher in people with DED than those without. Furthermore, when compared with other common ocular disorders, the dry eye feeling had the biggest effect on mental health. In a study in Beijing, Jonas et al. [44] examined all common ocular disorders and only dry eye feeling was linked to a higher depression score. Similarly, in a study conducted in the Netherlands, individuals with a highly symptomatic DED demonstrated the highest risk of a low SF-36 MCS score compared to other eye disorders [5]. At the population level, DED symptoms have been associated with depression or depressive symptoms also in the Beaver Dam Offspring Study and Korean DED prevalence study [3, 4]. Our results also indicated that a higher occurrence of subjective symptoms of DED (dryness feeling and/or OSDI) was associated with a higher depression score and a lower SF-36 MCS. This was the first study associating DED clinical signs with mental health at the population level. The conjunctival staining in women and blepharitis in men were associated with the depression score in our study population, implying that of ocular surface signs, ocular surface tissue defects rather than aqueous deficiencies tend to influence depression score more.

Non-response bias is a potential concern in all questionnaire-based studies, as individuals who did not complete all QoL questionnaire items may differ from those who did. However, missing QoL data did not appear to significantly impact our results, as sensitivity analysis of missing data supported our original findings. Additionally, our 15D results were in line with the Finnish Health 2011 population study, where the mean 15D score was 0.9 [45] and similar ceiling effects were observed in the same age group (Petri Purola, personal communication, Aug 19, 2024). The most notable difference between responders and nonresponders was the older age of the nonresponders. Given that DED is more prevalent in older individuals, the impact of DED on QoL may be underestimated in our SF-36 and BDI-II-related results due to this missing data. However, statistically significant differences in QoL between individuals with and without DED should not be confused with the change that a person perceives as beneficial or harmful. The generic minimum important change for the 15D score is ± 0.015, suggesting that the differences in the 15D score observed between DED and non-DED participants in our study can be clinically important [46]. While a difference of five or more points in SF-36 subscale scores and two to three points in BDI-II scores have been considered clinically relevant in other conditions or treatments [47, 48], our comparisons based on DED diagnosis did reveal possible clinically meaningful changes in BDI-II scores but not in SF-36 scores. Clinically important differences can also be context-specific [49].

This study was not without limitations. In future, we recommend that QoL research should use a broad age range to understand DED across different ages. Our study population was from rural community; ideally both urban and rural areas should be included to ensure broader applicability of the findings. A longitudinal design would be valuable for capturing the progression and impact of DED on QoL over time. The selection of confounding factors could be broadened with e.g. environmental and life-style factors. On basis of this research, we recommend a careful selection of the diagnostic criteria for DED to enhance consistency and comparability across studies. For DED-related HRQoL, to minimize the complexity we recommend using the 15D instrument. Additionally, a large sample size is essential to improve reliability and facilitate subgroup analysis, as DED signs, symptoms, and subjective QoL experiences of DED differ between women and men. We believe that our current results offer valuable insights for the future research on the underlying mechanisms of sex-differences in DED.

## Conclusions

Our analyses demonstrated a significant association between DED and a low QoL scores. Our findings indicated that DED notably increased the odds of lower mental health-related QoL (SF-36 MCS and BDI-II) and overall QoL (15D). Multiple analyses revealed that the subjective DED symptoms were statistically significantly associated with reduced QoL, particularly in women. For the first time at the population level, we showed that worse ocular surface signs correlated with worse QoL; NIBUT, corneal and conjunctival staining in all participants, NIBUT and conjunctival staining in women, and blepharitis in men. Our results also highlight the difference in how DED symptoms may affect the QoL in women and men. Therefore, a careful selection of diagnostic criteria, consideration of subjective symptoms, and an individualized approach to treating women and men with DED should be employed in clinical settings where DED is treated.

## Electronic supplementary material

Below is the link to the electronic supplementary material.

Supplementary Material 2

## References

[1] Pflugfelder, S. C., & Stern, M. (2020). Biological functions of tear film. *Experimental Eye Research*, *197*, 108115. 10.1016/j.exer.2020.108115 32561483 10.1016/j.exer.2020.108115 PMC7483968

[2] Ahn, J. M., Lee, S. H., Rim, T. H., Park, R. J., Yang, H. S., Kim, T. I., Yoon, K. C., & Seo, K. Y. (2014). *Epidemiologic Survey Committee of the Korean Ophthalmological Society. Prevalence of and risk factors associated with dry eye*. the Korea National Health and Nutrition Examination. 10.1016/j.ajo.2014.08.021 25149910

[3] Paulsen, A. J., Cruickshanks, K. J., Fischer, M. E., Huang, G. H., Klein, B. E., Klein, R., & Dalton, D. S. (2014). Dry eye in the beaver dam offspring study: Prevalence, risk factors, and health-related quality of life. *American Journal of Ophthalmology*, *157*(4), 799–806. 10.1016/j.ajo.2013.12.023 24388838 10.1016/j.ajo.2013.12.023 PMC3995164

[4] Dana, R., Meunier, J., Markowitz, J. T., Joseph, C., & Siffel, C. (2020). Patient-reported Burden of Dry Eye Disease in the United States: Results of an online cross-sectional survey. *American Journal of Ophthalmology*, *216*, 7–17. 10.1016/j.ajo.2020.03.044 32277941 10.1016/j.ajo.2020.03.044

[5] Morthen, M. K., Magno, M. S., Utheim, T. P., Snieder, H., Hammond, C. J., & Vehof, J. (2021). The physical and mental burden of dry eye disease: A large population-based study investigating the relationship with health-related quality of life and its determinants. *Ocular Surface*, *21*, 107–117. 10.1016/j.jtos.2021.05.006 34044135 10.1016/j.jtos.2021.05.006

[6] Lim, E. W. L., Chong, C. C. Y., Nusinovici, S., Fenwick, E., Lamoureux, E. L., Sabanayagam, C., Cheng, C. Y., & Tong, L. (2023). Relationship between dry eye symptoms and quality of life: Associations and mediation analysis. *British Journal of Ophthalmology*, *107*(11), 1606–1612. 10.1136/bjo-2022-321246 35940854 10.1136/bjo-2022-321246

[7] Münch, K., Nöhre, M., Westenberger, A., Akkus, D., Morfeld, M., Brähler, E., Framme, C., & de Zwaan, M. (2023). Prevalence and Correlates of Dry Eye in a German Population Sample. *Cornea*, Sep 14. 10.1097/ICO.0000000000003374Survey 2010–2011. *American Journal of Ophthalmology, 158*(6), 1205–1214.e7. 10.1016/j.ajo.2014.08.021. 10.1097/ICO.0000000000003374 PMC11073563 37713656

[8] Boboridis, K. G., Messmer, E. M., Benítez-Del-Castillo, J., Meunier, J., Sloesen, B., O’Brien, P., Quadrado, M. J., Rolando, M., & Labetoulle, M. (2023). Patient-reported burden and overall impact of dry eye disease across eight European countries: A cross-sectional web-based survey. *British Medical Journal Open*, *13*(3), e067007. 10.1136/bmjopen-2022-067007 10.1136/bmjopen-2022-067007 PMC10030789 36931668

[9] Kai, J. Y., Wu, Y. B., Shi, B., Li, D. L., Dong, X. X., Wang, P., & Pan, C. W. (2024). Dry eye symptoms and health-related quality of life among Chinese individuals: A national-based study. *British Journal of Ophthalmology*. 10.1136/bjo-2023-324677., bjo-2023-324677. 38471750 10.1136/bjo-2023-324677

[10] Stapleton, F., Alves, M., Bunya, V., Jalbert, I., Lekhanont, K., Malet, F., Na, K-S., Schaumberg, D., Uchino, M., Vehof, J., Viso, E., Vitale, S., & Jones, L. (2017). TFOS DEWS II Epidemiology report. *Ocular Surface*, *15*(3), 334–365. 10.1016/j.jtos.2017.05.003 28736337 10.1016/j.jtos.2017.05.003

[11] Kawashima, M., Yamada, M., Shigeyasu, C., Suwaki, K., Uchino, M., Hiratsuka, Y., Yokoi, N., Tsubota, K., & For The Decs-J FTD. (2020). Association of systemic comorbidities with dry eye disease. *Journal of Clinical Medicine, 9*(7), 2040. 10.3390/jcm9072040 10.3390/jcm9072040 PMC7408955 32610609

[12] Tsai, C. Y., Jiesisibieke, Z. L., & Tung, T. H. (2022). Association between dry eye disease and depression: An umbrella review. *Frontiers in Public Health*, *10*, 910608. 10.3389/fpubh.2022.910608 36466469 10.3389/fpubh.2022.910608 PMC9713230

[13] Basilious, A., Xu, C. Y., & Malvankar-Mehta, M. S. (2022). Dry eye disease and psychiatric disorders: A systematic review and meta-analysis. *European Journal of Ophthalmology*, *32*, 1872–1889. 10.1177/11206721211060963 34935549 10.1177/11206721211060963 PMC9297048

[14] McDonald, M., Patel, D. A., Keith, M. S., & Snedecor, S. J. (2016). Economic and humanistic burden of dry eye disease in Europe, North America, and Asia: A systematic literature review. *Ocular Surface*, *14*(2), 144–167. 10.1016/j.jtos.2015.11.002 26733111 10.1016/j.jtos.2015.11.002

[15] Okumura, Y., Inomata, T., Iwata, N., Sung, J., Fujimoto, K., Fujio, K., Midorikawa-Inomata, A., Miura, M., Akasaki, Y., & Murakami, A. (2020). A review of dry eye questionnaires: Measuring patient-reported outcomes and health-related quality of life. *Diagnostics*, *10*(8), 559. 10.3390/diagnostics10080559 32764273 10.3390/diagnostics10080559 PMC7459853

[16] Sánchez-Brau, M., Seguí-Crespo, M., Cantó-Sancho, N., Tauste, A., & Ramada, J. M. (2023). What are the dry eye questionnaires available in the scientific literature used for? A scoping review. *American Journal of Ophthalmology*, *246*, 174–191. 10.1016/j.ajo.2022.10.019 36336073 10.1016/j.ajo.2022.10.019

[17] Aapola, U., Nättinen, J., Suurkuukka, I., Tuomilehto, J., Keinänen-Kiukaanniemi, S., Saramies, J., & Uusitalo, H. (2022). Ocular surface health of the Finnish elderly population. *Acta Ophthalmologica*, *100*(8), 894–902. 10.1111/aos.15130 35322930 10.1111/aos.15130 PMC9790390

[18] Saramies, J., Koiranen, M., Auvinen, J., Uusitalo, H., Hussi, E., Cederberg, H., Keinänen-Kiukaanniemi, S., & Tuomilehto, J. (2021). 22-year trends in dysglycemia and body mass index: A population-based cohort study in Savitaipale, Finland. *Primary Care Diabetes*, *15*(6), 977–984. 10.1016/j.pcd.2021.09.010 34649826 10.1016/j.pcd.2021.09.010

[19] Saramies, J., Koiranen, M., Auvinen, J., Uusitalo, H., Hussi, E., Becker, S., Keinänen-Kiukaanniemi, S., Tuomilehto, J., & Suija, K. (2022). A natural history of erectile dysfunction in elderly men: A population-based twelve-year prospective study. *Journal of Clinical Medicine*, *11*(8), 2146. 10.3390/jcm11082146 35456238 10.3390/jcm11082146 PMC9029758

[20] Saunajoki, A., Auvinen, J., Bloigu, A., Saramies, J., Tuomilehto, J., Uusitalo, H., Hussi, E., Cederberg-Tamminen, H., Suija, K., Keinänen-Kiukaanniemi, S., & Timonen, M. (2022). Elevated one-hour post-load glucose in independently associated with albuminuria: A cross-sectional population study. *Journal of Clinical Medicine*, *11*(14), 4124. 10.3390/jcm11144124 35887888 10.3390/jcm11144124 PMC9317539

[21] von Elm, E., Altman, D. G., Egger, M., Pocock, S. J., Gøtzsche, P. C., Vandenbroucke, J. P., & STROBE Initiative. (2007). The strengthening the reporting of Observational studies in Epidemiology (STROBE) statement: Guidelines for reporting observational studies. *Lancet*, *370*(9596), 1453–1457. 10.1016/S0140-6736(07)61602-X 18064739 10.1016/S0140-6736(07)61602-X

[22] Sintonen, H. (2001). The 15D instrument of health-related quality of life: Properties and applications. *Annals of Medicine*, *33*, 328–336. 10.3109/07853890109002086 11491191 10.3109/07853890109002086

[23] Ware, J. E., & Sherbourne, C. D. (1992). The MOS 36-item short-form health survey (SF-36): I. conceptual framework and item selection. *Medical Care*, *30*, 473–483. 1593914

[24] Ware, J. E., Kosinski, M., & Keller, S. D. (1993). SF-36 physical and mental health summary scales: a user’s manual. Vol. 7. Boston. The Health Assessment Lab, New England Medical Center.

[25] Beck, A. T., Steer, R. A., & Brown, G. (1996). *Manual for the Beck Depression Inventory-Second Edition*. Psychological Corporation.

[26] Wolffsohn, J. S., Arita, R., Chalmers, R., Djalilian, A., Dogru, M., Dumbleton, K., Gupta, P. K., Karpecki, P., Lazreg, S., Pult, H., Sullivan, B. D., Tomlinson, A., Tong, L., Villani, E., Yoon, K. C., Jones, L., & Craig, J. P. (2017). TFOS DEWS II Diagnostic Methodology report. *Ocular Surface*, *15*(3), 539–574. 10.1016/j.jtos.2017.05.001 28736342 10.1016/j.jtos.2017.05.001

[27] Sullivan, D. A., Rocha, E. M., Aragona, P., Clayton, J. A., Ding, J., Golebiowski, B., Hampel, U., McDermott, A. M., Schaumberg, D. A., Srinivasan, S., Versura, P., & Willcox, M. D. P. (2017). TFOS DEWS II sex, gender, and hormones Report. *Ocular Surface*, *15*(3), 284–333. 10.1016/j.jtos.2017.04.001 28736336 10.1016/j.jtos.2017.04.001

[28] Matossian, C., McDonald, M., Donaldson, K. E., Nichols, K. K., MacIver, S., & Gupta, P. K. (2019). Dry Eye Disease: Consideration for women’s Health. *Journal of Women’s Health (Larchmt)*, *28*(4), 502–514. 10.1089/jwh.2018.7041 10.1089/jwh.2018.7041 PMC6482917 30694724

[29] Vehof, J., Smitt-Kamminga, N. S., Nibourg, S. A., & Hammond, C. J. (2018). Sex differences in clinical characteristics of dry eye disease. *Ocular Surface*, *16*, 242–248. 10.1016/j.jtos.2018.01.001 29317327 10.1016/j.jtos.2018.01.001

[30] Noland, S. T., Badian, R. A., Utheim, T. P., Utheim, O. A., Stojanovic, A., Tashbayev, B., Raeder, S., Dartt, D. A., & Chen, X. (2021). Sex and age differences in symptoms and signs of dry eye disease in a Norwegian cohort of patients. *Ocular Surface*, *19*, 68–73. 10.1016/j.jtos.2020.11.009 33246035 10.1016/j.jtos.2020.11.009

[31] Borrelli, M., Fings, A., Geerling, G., & Finis, D. (2021). Gender-specific differences in signs and symptoms of dry eye disease. *Current Eye Research*, *46*(3), 294–301. 10.1080/02713683.2020.1801758 32735461 10.1080/02713683.2020.1801758

[32] Zhao, M., Yu, Y., Roy, N. S., Ying, G. S., Asbell, P., & Bunya, V. Y. (2023). Sex-related differences and hormonal effects in the Dry Eye Assessment and Management (DREAM) study. *British Journal of Ophthalmology*, *108*(1), 23–29. 10.1136/bjo-2022-322238 36575626 10.1136/bjo-2022-322238 PMC10285651

[33] Le, Q., Zhou, X., Ge, L., Wu, L., Hong, J., & Xu, J. (2012). Impact of dry eye syndrome on vision-related quality of life in a non-clinic-based general population. *BMC Ophthalmology*, *12*, 22. 10.1186/1471-2415-12-22 22799274 10.1186/1471-2415-12-22 PMC3437197

[34] Morthen, M. K., Magno, M. S., Utheim, T. P., Snieder, H., Jansonius, N., Hammond, C. J., & Vehof, J. (2022). The vision-related burden of dry eye. *The Ocular Surface*, *23*, 207–215. 10.1016/j.jtos.2021.10.007 34743866 10.1016/j.jtos.2021.10.007

[35] Belenguer, R., Ramos-Casals, M., Brito-Zerón, P., del Pino, J., Sentís, J., Aguiló, S., & Font, J. (2005). Influence of clinical and immunological parameters on the health-related quality of life of patients with primary Sjögren’s syndrome. *Clinical and Experimental Rheumatology*, *23*(3), 351–356. 15971423

[36] Mizuno, Y., Yamada, M., Miyake, Y., & Dry Eye Survey Group of the National Hospital Organization of Japan. (2010). Association between clinical diagnostic tests and health-related quality of life surveys in patients with dry eye syndrome. *Japanese Journal of Ophthalmology*, *54*(4), 259–265. 10.1007/s10384-010-0812-2 20700790 10.1007/s10384-010-0812-2

[37] Wang, T. J., Wang, I. J., Hu, C. C., & Lin, H. C. (2012). Comorbidities of dry eye disease: A nationwide population-based study. *Acta Ophthalmologica*, *90*(7), 663–668. 10.1111/j.1755-3768.2010.01993.x 20809911 10.1111/j.1755-3768.2010.01993.x

[38] Roh, H. C., Lee, J. K., Kim, M., Oh, J. H., Chang, M. W., Chuck, R. S., & Park, C. Y. (2016). Systemic comorbidities of Dry Eye Syndrome: The Korean National Health and Nutrition Examination Survey V, 2010 to 2012. *Cornea*, *35*(2), 187–192. 10.1097/ICO.0000000000000657 26488632 10.1097/ICO.0000000000000657

[39] Dana, R., Bradley, J. L., Guerin, A., Pivneva, I., Evans, A. M., & Stillman, I. Ö. (2019). Comorbidities and prescribed medications in patients with or without Dry Eye Disease: A Population-based study. *American Journal of Ophthalmology*, *198*, 181–192. 10.1016/j.ajo.2018.10.001 30312577 10.1016/j.ajo.2018.10.001

[40] Yu, K., Bunya, V., Maguire, M., Asbell, P., & Ying, G. S. (2021). Dry Eye Assessment and Management Study Research Group. Systemic conditions Associated with Severity of Dry Eye signs and symptoms in the Dry Eye Assessment and Management Study. *Ophthalmology*, *128*(10), 1384–1392. 10.1016/j.ophtha.2021.03.030 33785415 10.1016/j.ophtha.2021.03.030 PMC8463420

[41] Guo, M., Diaz, G. M., Yu, Y., Patel, C. A., Farrar, J. T., Asbell, P. A., Ying, G. S., & Management Study Research Group. (2024). Dry Eye Assessment and. Association between systemic medication use and severity of dry eye signs and symptoms in the DRy eye assessment and management (DREAM) study. *Ocular Surface 32*, 112–119. 10.1016/j.jtos.2024.01.009 10.1016/j.jtos.2024.01.009 PMC11056304 38307463

[42] Bartlett, J. D., Keith, M. S., Sudharshan, L., & Snedecor, S. J. (2015). Associations between sign and symptoms of dry eye disease: A systematic review. *Clinical Ophthalmology*, *9*, 1719–1730. 10.2147/OPTH.S89700 26396495 10.2147/OPTH.S89700 PMC4577273

[43] Wan, K. H., Chen, L. J., & Young, A. L. (2016). Depression and anxiety in dry eye disease: A systematic review and meta-analysis. *Eye (Lond)*, *30*(12), 1558–1567. 10.1038/eye.2016.186 27518547 10.1038/eye.2016.186 PMC5177754

[44] Jonas, J. B., Wei, W. B., Xu, L., Rietschel, M., Streit, F., & Wang, Y. X. (2018). Self-rated depression and eye diseases: The Beijing Eye Study. *PLoS One*, *13*(8), e0202132. 10.1371/journal.pone.020213 30096194 10.1371/journal.pone.0202132 PMC6086446

[45] Koskinen, S., Lundqvist, A., & Ristiluoma, N. (Eds.). (2012). eds. Health, functional capacity and welfare in Finland in 2011. National Institute for Health and Welfare (THL), Report 68/2012. 290 pages. Helsinki 2012. ISBN 978-952-245- 768-4 (printed), ISBN 978-952-245-769-1 (online publication).

[46] Alanne, S., Roine, R. P., Räsänen, P., Vainiola, T., & Sintonen, H. (2015). Estimating the minimum important change in the 15D scores. *Quality of Life Research*, *24*(3), 599–606. 10.1007/s11136-014-0787-4 25145637 10.1007/s11136-014-0787-4

[47] Ware, J. E., Snow, M. S., Kosinski, M. A., & Gandek, M. S. (1993). *SF-36 Health Survey Manual and Interpretation Guide*. The Health Institute, New England Medical Centre.

[48] NCCMH. (2004). *Depression: Management of Depression in primary and secondary care*. British Psychological Society and Royal College of Psychiatrists: Leicester and London [full guideline].

[49] Wang, Y. C., Hart, D. L., Stratford, P. W., & Mioduski, J. E. (2011). Baseline dependency of minimal clinically important improvement. *Physical Therapy & Rehabilitation Journal*, *91*(5), 675–688. 10.2522/ptj.20100229 21372203 10.2522/ptj.20100229

