# Supplementary material for: Impact of signs and symptoms of dry eye disease on health-related quality of life: a cross-sectional population study among older adults
Source: Qual Life Res. 2025 Jan 28;34(5):1363–76. doi: 10.1007/s11136-025-03907-0 (PMC12064583; doi:10.1007/s11136-025-03907-0)
Supplement: Supplementary file 1 — Supplementary Material 2 [file 11136_2025_3907_MOESM2_ESM.pdf]

Quality of Life Research - journal: Impact of signs and symptoms of dry eye disease on health-related quality of life: a cross-sectional population study among older adults

Ulla Aapola, Paula Mosallaei, Janika Nättinen, Ilona Suurkuukka, Jaakko Tuomilehto, Sirkka Keinänen-Kiukaanniemi, Jouko Saramies, Hannu Uusitalo

Corresponding author: Ulla Aapola, Eye and Vision Research, Faculty of Medicine and Health Technology, Tampere University, Tampere, Finland, email: ulla.aapola@tuni.fi

Online Resource 1 The prevalence of diagnoses categorized into nine comorbidity groups in the studied population

Comorbidity group	ICD-10 Codes	All positive N (%)	All negative N (%)	Women positive N (%)	Women negative N (%)	Men positive N (%)	Men negative N (%)
Cancer	C00-C97, D10-D48	178 (30)	281 (47)	98 (29)	158 (47)	80 (30)	123 (46)
Connective tissue diseases	M05-M07, M30-M35, M45	28 (5)	431 (72)	15 (4)	241 (72)	13 (5)	190(71)
Diabetes	E10-E14	56 (9)	403 (67)	26 (8)	230 (67)	30 (11)	173 (65)
Heart diseases	I20-I28, I30-I52, Q20-Q24	178 (30)	281 (47)	90 (27)	166 (50)	88 (33)	115 (43)
Hypertension	I10-I14	160 (27)	299 (50)	89 (27)	167 (50)	71 (27)	132 (50)
Musculoskeletal conditions	M00-M14, M20-M25, M46-M54, M60-M63, M65-M68, M70-M99, S02, S12, S22, S32, S42, S52, S62, S72, S82, S92	273 (45)	186 (31)	158 (47)	98 (29)	115 (43)	88 (33)
Psychiatric diseases	F10-F48, F50-F62, F80-F89, F99	44 (7)	415 (69)	25 (7)	231 (69)	19 (7)	184 (69)
Pulmonary diseases	A15-A19, J40-J47, J60-J70, J80-J86, J90-J94, J96, J98, J99	44 (7)	415 (69)	21 (6)	235 (70)	23 (9)	180 (68)
Vascular diseases	I15, I60-I89, I95-99, Q25-Q28	124 (21)	335 (56)	67 (20)	189 (56)	57 (21)	146 (55)

The percentage of the total population (601 for total, 335 for women, and 266 for men), is given in the parentheses.

Online Resource 2 Nature of missing data and sensitivity analyses evaluating the potential impact of missing data (pages 2-6)

Potential impact of missing QoL data on our results was assessed through several stages of sensitivity analysis. We first examined the missing data patterns to determine if the missing values were Missing Completely at Random (MCAR), Missing at Random (MAR) or Missing Not at Random (MNAR). MCAR test in R, adjusted using the Benjamini-Hochberg correction, revealed that none of the QoL dimensions or summary scores were MCAR (adjusted p-values << 0.05). Due to lack of a follow-up, it was not possible to differentiate between MAR and MNAR for the missing data. Since our data were not MCAR we performed an imputation for 15D data as instructed by the developer of the 15D questionnaire (www.15D-instrument.net). For SF-36, the imputation was only possible for 13 respondents in the Energy/Fatigue dimension, as per Ware et al. 1993 [22], leading to eight additional summary scores. No imputation was performed for BDI-II, as no guidelines are provided by its creators. After the imputation, the missing values constituted merely 4 % of the 15D data. As a result, we excluded this questionnaire from the further sensitivity analysis and focused on analyzing SF36 PCS and MCS, and BDI-II.

To investigate if missingness in QoL values depended on other variables, we compared the age, sex, clinical signs, medication use, and OSDI between SF36 and BDI-II responders and nonresponders. Continuous variables were tested using Wilcoxon rank sum test, while ordinal and categorical variables were tested with Fisher's exact test due to low categorical frequencies (< 5). The results are presented in the table below.

Differences in outcomes between responders and nonresponders

**Ques-tion-naire**	**Variable, mean** (SD) **or median** (interquartile range)	**Responder**	**Non-responder**	**p-value**	**Group**
SF-36	Age	70.7 (5.9)	75.3 (6.2)	<0.001	All
		70.9 (5.9)	75.9 (5.9)	<0.001	Women
		70.5 (5.8)	74.5 (6.5)	<0.001	Men
	BCVA	0.89 (0.14)	0.84 (0.19)	<0.001	All
		0.88 (0.15)	0.83 (0.18)	0.01	Women
		0.91 (0.12)	0.85 (0.19)	0.005	Men
	NIBUT	9.0 (1.9)	8.4 (2.5)	0.03	Women
	Medication frequency	0 (0-1)	0 (0-1)	0.018	All
		0 (0-0)	0 (0-0)	0.03	Men
BDI-II	Age	71.0 (6.0)	75.6 (6.9)	<0.001	All
		71.2 (6.1)	75.6 (5.9)	<0.001	Women
		70.8 (6.0)	75.5 (6.2)	<0.001	Men
	BCVA	0.90 (0.14)	0.81 (0.19)	<0.001	All
		0.88 (0.15)	0.81 (0.18)	0.003	Women
		0.91 (0.12)	0.81 (0.21)	<0.001	Men
	NIBUT	9.1 (1.9)	8.5 (2.3)	0.07	All
		9.1 (1.9)	8.2 (2.4)	<0.001	Women

In addition, we examined the association between the missingness and other variables through logistic regression, in which the dependent variable was a dichotomous variable describing the missingness, and the independent variables were age, sex, clinical values, medication use, and OSDI. The significant predictors can be seen in the table below, with p-values adjusted using Benjamini-Hochberg correction.

**Questionnaire**	**Variable**	**Coefficient**	**P-value**	**Adjusted p-value**
SF-36	Age	0.117	< 0.001	< 0.001
BDI-II	Age	0.098	< 0.001	< 0.001

In both analyses, age was the most significant variable associated with the occurrence of missing data. Nonresponders were consistently older than responders in both sexes and both questionnaires. Although, there were also minor differences in NIBUT, BCVA, OSDI and corneal staining results, these differences, while statistically significant, lacked clear clinical relevance. Furthermore, it is well-established that age influences these ocular examination results as evidenced also by our findings: nonresponders consistently had worse outcomes compared with responders.

Next, we conducted best-case and worst-case scenario analyses by age- and sex-matching nonresponders with responders. We imputed the highest and lowest observed QoL summary scores to create the hypothetical best-case and worst-case scenarios, respectively. We repeated the original analysis steps, including (1) correlation analysis between QoL summary scores and ophthalmological results, (2) comparison of hypothetical QoL mean scores across three DED diagnoses (DE, OSD, OSDI), (3) odds ratio (OR) analysis of low HRQoL or high BDI-II scores in people with and without DED, and (4) hierarchical linear regression (HLR) with best- and worst-case QoL values as dependent variables.

The correlation analysis **(step 1)** showed that the direction and significance of the correlation was either consistent with the original analysis or became non-significant. Two variables that had a significant correlation in the original analysis reversed direction in the hypothetical best-case scenarios, but correlations were not significant (BCVA of all in BDI-II and NIBUT of women in SF-36 MCS) as seen in correlation tables below.

The correlation between the ocular surface signs or symptoms and the QoL scores performed with hypothetical best-case and worst-case values imputed to missing QoL data for all, women and men. BC=best-case results, WC=worst-case results. Green: significant and consistent direction of correlation compared to the original result. Orange: non-significant and consistent direction of correlation compared to original result. Pink: non-significant and non-consistent direction of correlation compared to original result. Grey: a newly found significant correlation.

		ALL, Correlation coefficient<br>Adjusted P – value								
		OSDI	Blepharitis	MGD	Conjunctival redness	Corneal staining	Conjunctival staining	NIBUT	Schirmer test	BCVA
SF-36 PCS	BC n=601	-0,13	0,01	0,00	-0,05	0,00	-0,03	0,01	-0,03	-0,06
		0,009	0,851	0,946	0,379	0,946	0,646	0,801	0,607	0,249
	WC n=601	-0,042	-0,02	0,08	-0,065	0,031	-0,071	0,120	-0,066	0,177
		0,448	0,759	0,100	0,218	0,585	0,165	0,012	0,211	0,000
SF-36 MCS	BC n=601	-0,12	-0,03	-0,05	-0,04	-0,05	-0,03	0,03	0,01	-0,10
		0,012	0,575	0,345	0,470	0,352	0,593	0,650	0,851	0,053
	WC n=601	-0,033	-0,060	0,029	-0,054	-0,019	-0,075	0,129	-0,038	0,157
		0,574	0,256	0,607	0,296	0,759	0,146	0,007	0,493	0,001
BDI-II	BC n=601	0,13	0,02	0,00	0,01	0,06	0,07	-0,03	0,03	0,01
		0,009	0,677	0,997	0,864	0,263	0,156	0,583	0,637	0,801
	WC n=601	0,106	0,074	0,018	0,076	0,060	0,151	-0,156	-0,016	-0,240
		0,030	0,152	0,764	0,142	0,256	0,001	0,001	0,784	0,000

		WOMEN, Correlation coefficient<br>Adjusted P – value								
		OSDI	Blepharitis	MGD	Conjunctival redness	Corneal staining	Conjunctival staining	NIBUT	Schirmer test	BCVA
SF-36 PCS	BC n=335	-0,16	0,06	0,03	0,01	0,03	-0,01	-0,04	0,01	-0,06
		0,015	0,485	0,795	0,929	0,762	0,925	0,698	0,924	0,475
	WC n=335	-0,06	0,03	0,09	-0,05	0,07	-0,07	0,16	-0,04	0,15
		0,449	0,746	0,249	0,519	0,370	0,370	0,015	0,633	0,035
SF-36 MCS	BC n=335	-0,12	-0,04	0,01	-0,02	-0,06	-0,03	-0,01	0,04	-0,10
		0,106	0,634	0,935	0,850	0,498	0,762	0,935	0,632	0,224
	WC n=335	-0,02	-0,07	0,07	-0,08	-0,01	-0,08	0,19	-0,02	0,12
		0,830	0,370	0,370	0,332	0,924	0,300	0,004	0,830	0,094
BDI-II	BC n=335	0,14	-0,01	-0,03	0,01	0,04	0,12	0,00	0,02	0,01
		0,036	0,914	0,760	0,935	0,650	0,110	0,987	0,806	0,924
	WC n=335	0,06	0,05	-0,01	0,09	0,07	0,18	-0,20	-0,07	-0,20
		0,498	0,585	0,924	0,292	0,370	0,008	0,002	0,421	0,002

		MEN, Correlation coefficient<br>Adjusted P – value								
		OSDI	Blepharitis	MGD	Conjunctival redness	Corneal staining	Conjunctival staining	NIBUT	Schirmer test	BCVA
SF-36 PCS	BC n=266	-0,07	-0,01	0,00	-0,09	-0,07	-0,07	0,10	-0,10	-0,06
		0,435	0,909	0,970	0,320	0,435	0,432	0,261	0,290	0,489
	WC n=266	0,01	-0,13	0,05	-0,12	0,00	-0,06	0,05	-0,09	0,22
		0,926	0,215	0,560	0,222	0,996	0,498	0,515	0,298	0,008
SF-36 MCS	BC n=266	-0,10	-0,01	-0,12	-0,08	-0,04	-0,05	0,07	-0,03	-0,11
		0,261	0,901	0,217	0,377	0,598	0,560	0,437	0,730	0,232
	WC n=266	-0,01	-0,12	-0,06	-0,11	0,02	-0,03	0,03	-0,05	0,19
		0,896	0,217	0,461	0,249	0,784	0,708	0,730	0,560	0,027
BDI-II	BC n=266	0,08	0,09	0,07	0,05	0,07	0,00	-0,07	0,02	0,03
		0,377	0,298	0,435	0,543	0,435	0,996	0,435	0,801	0,730
	WC n=266	0,11	0,20	0,12	0,17	-0,02	0,09	-0,05	0,03	-0,28
		0,230	0,023	0,217	0,068	0,806	0,304	0,575	0,730	0,000

Correlation coefficients were calculated for each individual variable using Spearman correlation for ordinal variables and Pearson correlation for continuous variables. OSDI=ocular surface disease index; MGD=meibomian gland disease, NIBUT=noninvasive tear film break-up time, BCVA=best corrected visual acuity, N = number of participants, PCS=physical component summary, MCS=mental component summary, BDI=Beck's depression inventory

Comparison of the QoL mean scores in people with DED diagnosis and without it **(step 2)** supported our initial conclusion that people with DED diagnosis had worse QoL scores than people without DED. DE diagnosis criteria were the most sensitive, and OSDI diagnosis criteria were the least sensitive to changes in QoL score values as indicated in table below.

The quality of life (QoL) mean scores of dry eye positive and negative participants according to different diagnostic categories (OSDI, DE, OSD) performed with hypothetical best-case and worse-case values imputed to missing QoL data for all, women and men.

		All, mean score (SD)			Women, mean score (SD)			Men, mean score (SD)		
Diagnosis category	QoL questionnaire	Pos[1]	Neg	p-val	Pos	Neg	p-val	Pos	Neg	p-val
OSDI[2]	SF-36 PCS original	**44.0 (9.9)**	**46.7 (9.3)**	**0.021**	**43.0 (10.1)**	**47.0 (9.7)**	**0.006**	46.8 (8.7)	46.4 (8.9)	0.922
	Best Case	49.1 (11.8)	51.3 (10.5)	0.132	**48.5 (12.5)**	**52.0 (11.1)**	**0.026**	50.9 (9.7)	50.5 (9.9)	0.794
	Worst Case	37.1 (14.1)	38.4 (14.1)	0.213	35.5 (13.9)	38.3 (15.4)	0.270	38.6 (14.8)	38.4 (14.6)	0.984
	SF-36 MCS original	**51.3 (10.1)**	**54.6 (9.2)**	**<0.001**	**50.9 (10.5)**	**53.9 (9.4)**	**0.023**	52.4 (9.2)	55.4 (9.0)	0.051
	Best Case	**56.7 (12.3)**	**59.5 (10.7)**	**0.035**	56.3 (12.7)	59.1 (11.1)	0.057	57.8 (11.5)	59.9 (10.3)	0.244
	Worst Case	**43.5 (15.3)**	**45.4 (16.1)**	**0.046**	43.2 (15.6)	44.2 (16.5)	0.399	44.4 (14.7)	46.7 (15.7)	0.224
	BDI-II original	**7.6 (5.9)**	**5.3 (5.1)**	**<0.001**	**7.6 (5.9)**	**5.8 (4.8)**	**0.011**	**7.4 (6.2)**	**4.9 (5.2)**	**0.025**
	Best Case	**5.7 (6.1)**	**4.1 (5.0)**	**0.009**	**5.8 (6.1)**	**4.2 (13.3)**	**0.050**	5.7 (6.3)	4.0 (5.1)	0.155
	Worst Case	**13.4 (11.6)**	**11.2 (11.7)**	**0.003**	13.9 (12.1)	13.3 (12.9)	0.215	**12.0 (10.0)**	**9.0 (9.8)**	**0.041**
DE[3]	SF-36 PCS original	44.9 (9.8)	46.3 (9.4)	0.436	44.5 (10.1)	46.1 (10.0)	0.403	45.9 (9.5)	46.5 (8.8)	0.928
	Best Case	49.8 (11.7)	50.9 (10.7)	0.722	50.6 (12.2)	51.0 (11.5)	0.941	47.1 (10.0)	50.8 (9.8)	0.143
	Worst Case	37.8 (14.4)	38.2 (14.8)	0.555	36.1 (14.7)	38.2 (15.0)	0.360	43.9 (11.8)	38.1 (14.7)	0.182
	SF-36 MCS original	**51.9 (9.1)**	**54.1 (9.5)**	**0.029**	51.3 (10.0)	53.3 (9.7)	0.167	53.4 (6.5)	55.1 (9.2)	0.163
	Best Case	57.1 (11.6)	59.1 (11.1)	0.233	57.8 (12.4)	58.4 (11.5)	0.709	**54.7 (7.9)**	**59.9 (10.5)**	**0.018**
	Worst Case	43.9 (15.3)	45.2 (16.0)	0.115	41.9 (16.0)	44.4 (16.2)	0.206	51.2 (10.0)	46.0 (15.8)	0.657
	BDI-II original	**7.6 (6.1)**	**5.6 (5.2)**	**0.011**	7.9 (6.2)	6.1 (5.0)	0.052	6.5 (5.7)	5.1 (5.4)	0.307
	Best Case	5.8 (6.2)	4.3 (5.1)	0.069	5.7 (6.4)	4.5 (5.0)	0.301	6.0 (5.8)	4.1 (5.2)	0.140
	Worst Case	**13.5 (12.0)**	**11.4 (11.6)**	**0.040**	15.0 (12.6)	13.1 (12.6)	0.129	8.1 (7.9)	9.5 (10.0)	0.982
OSD[4]	SF-36 PCS original	**44.4 (10.4)**	**47.0 (8.8)**	**0.016**	**44.0 (10.9)**	**47.0 (9.2)**	**0.038**	45.1 (9.6)	47.0 (8.4)	0.202
	Best Case	**49.0 (11.9)**	**51.6 (10.2)**	**0.028**	**48.8 (12.6)**	**52.0 (10.8)**	**0.036**	49.3 (10.8)	51.1 (9.4)	0.261
	Worst Case	37.9 (14.4)	38.4 (14.9)	0.560	37.8 (14.5)	38.4 (15.1)	0.723	38.1 (14.3)	38.5 (14.7)	0.740
	SF-36 MCS original	**52.2 (10.3)**	**54.8 (8.9)**	**0.006**	51.6 (10.5)	53.9 (9.2)	0.078	53.0 (9.9)	55.8 (8.6)	0.065
	Best Case	**57.0 (12.1)**	**59.7 (10.5)**	**0.012**	**56.5 (12.4)**	**59.2 (11.0)**	**0.047**	57.8 (11.6)	60.4 (10.0)	0.108
	Worst Case	44.9 (15.4)	45.4 (16.2)	0.386	44.4 (15.6)	44.2 (16.5)	0.843	45.6 (15.0)	46.6 (15.8)	0.498

BDI-II original	**6.8 (6.0)**	**5.3 (4.9)**	**0.022**	**7.3 (5.6)**	**5.8 (4.9)**	**0.021**	6.0 (6.5)	4.9 (4.9)	0.577
Best Case	4.8 (5.93)	4.3 (4.91)	0.864	5.0 (5.75)	4.5 (4.96)	0.881	4.6 (6.25)	4.0 (4.85)	0.904
Worst Case	**13.8 (12.3)**	**10.5 (11.1)**	**<0.001**	**15.6 (12.9)**	**11.9 (12.2)**	**0.003**	10.8 (10.5)	8.9 (9.6)	0.304

[1]Pos indicates positive dry eye diagnosis and Neg negative dry eye diagnosis, women (W) and men (M). Statistically significant results (Wilcoxon rank sum test, p-val < 0.05) are in bold. [2]Positive = OSDI>13; [3]Positive = OSDI>13 and at least one sign: NIBUT <10, corneal or conjunctival staining >0; [4]Positive = at least two signs or symptoms: NIBUT <10, corneal or conjunctival staining >0, Schirmer test <10 or OSDI>13. Number of participants in each QoL questionnaire data analysis is presented in Table 1. SD=standard deviation, OSDI=ocular surface disease index, DE=dry eye, OSD=ocular surface disease, PCS=physical component summary, MCS=mental component summary, BDI=Beck's depression inventory

The OR analysis **(step 3)** results can be seen in the table below. SF36-PCS and BDI-II scores as well as OSDI diagnosis criteria were the least sensitive to the changes. Again, DE diagnosis criteria were the most sensitive to the changes. OSDI and OSD diagnoses were in keeping with our original conclusion, that DED diagnosis imposes an increased risk of worsened mental health.

	OSDI OR (95% CI)	DE OR (95% CI)	OSD OR (95% CI)
**BEST CASE SCENARIO**			
SF-36 PCS	1.32 (0.87, 2.01)	1.08 (0.61, 1.88)	1.40 (0.97, 2.00)
SF-36 MCS	**1.77 (1.15, 2.73) ****	1.61 (0.91, 2.87)	**1.58 (1.10, 2.20) ***
BDI-II	**1.58 (1.04, 2.41) ***	1.52 (0.87, 2.65)	0.96 (0.67, 1.37)
**WORST CASE SCENARIO**			
SF-36 PCS	0.95 (0.59, 1.51)	0.86 (0.46, 1.62)	1.00 (0.66, 1.48)
SF-36 MCS	1.41 (0.91, 2.20)	1.42 (0.79, 2.56)	1.26 (0.86, 1.83)
BDI-II	**1.98 (1.26, 3.12) ****	1.73 (0.94, 3.18)	**1.82 (1.23, 2.67) ****

QoL scores were dichotomized based on the median score. OR was adjusted for age, sex, BCVA and comorbidities, 95% confidence intervals are in the parentheses, and statistical significance (Wald's test) is denoted by asterisks: * p-value < 0.05, ** p-value < 0.01, *** p-value < 0.001. DED=dry eye disease, OSDI=ocular surface disease index, DE=dry eye, OSD=ocular surface disease, BCVA=best corrected visual acuity, CI=confidence interval, PCS=physical component summary, MCS=mental component summary, BDI=Beck's depression inventory. DED diagnostic criteria can be found in Table 3.

HLR minimal model and a model with clinical values for the best-case scenario confirmed our previous finding of OSDI as the most significant variable affecting QoL in all participants and in women across all QoL scores (data not shown). The effect of OSDI on the QoL remained negative. HLR for the worst-case scenario did not confirm the OSDI finding but revealed that age was the most significant variable across all QoL scores, all models, and all studied subgroups, having an inverse effect on the QoL score. In addition, sex was significant across all models for BDI-II score, when all participants were considered, with female sex being a predictor of worse BDI-II.

Our sensitivity analysis suggested that missing data in our study was age-related, with older patients being less likely to complete questionnaires. Results could vary if the wellbeing of the oldest participants were as low as the lowest levels observed in our dataset. However, most of our original findings were robust despite missing QoL values.

Online Resource 3 Reliability and validity of QoL scores (pages 7-8)

The reliability of the QoL data was investigated through floor and ceiling effects, and through internal consistency. Internal validity of QoL scores was investigated with correlation analysis.

No floor effect but a clear ceiling effect was detected for 15D items ranging from 35 % (Discomfort and symptoms) to 99 % (Eating). Five out of eight dimensions of SF-36 reached the ceiling, ranging from 18 % (Physical functioning) to 72 % (Emotional well-being). A floor effect (20 %) was detected in one SF-36 dimension (Role physical). Distribution of BDI-II item scores were skewed to the lower points (no depression). Ceiling values of BDI-II varied between 32 % (no change in interest in sex) to 96 % (not feeling guilty). A floor effect of 16 % was detected for one BDI-II item (lost interested in sex completely). Interval consistencies and the validity of QoL scores are presented in tables below.

Reliability (internal consistency) of QoL scores

	**Internal consistency coefficients***			
SF36 PCS	0.92			
SF36 MCS	0.88			
	**Cronbach's alpha**	**2.5 % CI**	**97.5 % CI**	**Internal consistency**
SF36 Physical functioning	0.92	0.91	0.94	Excellent
SF36 Role lim physical health	0.90	0.88	0.92	Excellent
SF36 Role lim emotional health	0.86	0.82	0.89	Good
SF36 Energy/fatigue	0.75	0.70	0.80	Acceptable
SF36 Emotional wellbeing	0.77	0.72	0.81	Acceptable
SF36 Social functioning	0.80	0.75	0.85	Good
SF36 Pain	0.88	0.86	0.91	Good
SF36 General health	0.60	0.53	0.65	Questionable
15D Index	0.82	0.79	0.84	Good
BDI-II	0.85	0.82	0.88	Good

*Internal consistency coefficients of SF-36 PCS and MCS scores were calculated according to the method described in Ware et al, 1993, and they were in line with the coefficients from other studies presented in the publication.. Criteria chosen to evaluate Cronbach's alpha: <0.5 unacceptable; >= 0.5 and < 0.6 poor; >= 0.6 and < 0.7 questionable; >=0.7 < 0.8 acceptable; >=0.8 and < 0.9 good; >= 0.9 excellent.

Internal validity of QoL scores evaluated using correlation between the total summary score and individual dimensions/items.

	**Correlation coefficient**	**P-value**	**Adjusted p-value**	**Effect size**
SF36 PCS and SF36 MCS*	0.02**	0.723	-	Weak
**SF36 dimensions' correlation with SF36 PCS****				
SF36 Physical functioning	0.85	< 0.001	< 0.001	Strong
SF36 Role lim physical health	0.76	< 0.001	< 0.001	Strong
SF36 Role lim emotional health	0.30	< 0.001	< 0.001	Moderate
SF36 Energy/fatigue	0.41	< 0.001	< 0.001	Moderate
SF36 Emotional wellbeing	0.18	< 0.001	< 0.001	Weak
SF36 Social functioning	0.47	< 0.001	< 0.001	Moderate
SF36 Pain	0.68	< 0.001	< 0.001	Strong
SF36 General health	0.65	< 0.001	< 0.001	Strong

**SF36 dimensions' correlation with SF36 MCS****				
SF36 Physical functioning	0.13	0.008	0.008	Weak
SF36 Role lim physical health	0.22	< 0.001	< 0.001	Weak
SF36 Role lim emotional health	0.66	< 0.001	< 0.001	Strong
SF36 Energy/fatigue	0.72	< 0.001	< 0.001	Strong
SF36 Emotional wellbeing	0.82	< 0.001	< 0.001	Strong
SF36 Social functioning	0.61	< 0.001	< 0.001	Strong
SF36 Pain	0.24	< 0.001	< 0.001	Weak
SF36 General health	0.37	< 0.001	< 0.001	Moderate
**BDI-II items' correlation with BDI-II total score*****				
Item 1	0.36	< 0.001	< 0.001	Moderate
Item 2	0.49	< 0.001	< 0.001	Moderate
Item 3	0.47	< 0.001	< 0.001	Moderate
Item 4	0.45	< 0.001	< 0.001	Moderate
Item 5	0.28	< 0.001	< 0.001	Weak
Item 6	0.35	< 0.001	< 0.001	Moderate
Item 7	0.32	< 0.001	< 0.001	Moderate
Item 8	0.47	< 0.001	< 0.001	Moderate
Item 9	0.28	< 0.001	< 0.001	Weak
Item 10	0.33	< 0.001	< 0.001	Moderate
Item 11	0.45	< 0.001	< 0.001	Moderate
Item 12	0.46	< 0.001	< 0.001	Moderate
Item 13	0.52	< 0.001	< 0.001	Strong
Item 14	0.51	< 0.001	< 0.001	Strong
Item 15	0.68	< 0.001	< 0.001	Strong
Item 16	0.60	< 0.001	< 0.001	Strong
Item 17	0.69	< 0.001	< 0.001	Strong
Item 18	0.36	< 0.001	< 0.001	Moderate
Item 19	0.25	< 0.001	< 0.001	Weak
Item 20	0.47	< 0.001	< 0.001	Moderate
Item 21	0.61	< 0.001	< 0.001	Strong
**15D items' correlation with 15D Index*****				
Mobility	0.62	< 0.001	< 0.001	Strong
Vision	0.41	< 0.001	< 0.001	Moderate
Hearing	0.30	< 0.001	< 0.001	Moderate
Breathing	0.61	< 0.001	< 0.001	Strong
Sleeping	0.52	< 0.001	< 0.001	Strong
Eating	0.26	< 0.001	< 0.001	Weak
Talking	0.40	< 0.001	< 0.001	Moderate
Excretion	0.54	< 0.001	< 0.001	Strong
Usual activities	0.74	< 0.001	< 0.001	Strong
Mental functions	0.58	< 0.001	< 0.001	Strong
Discomfort and symptoms	0.66	< 0.001	< 0.001	Strong
Depression	0.47	< 0.001	< 0.001	Moderate
Distress	0.47	< 0.001	< 0.001	Moderate
Vitality	0.69	< 0.001	< 0.001	Strong
Sexual activity	0.53	< 0.001	< 0.001	Strong

*Correlation between the two summary scores of SF36. According to Ware et al. 1993 [22], the correlation between the PCS and MCS score should be very low, as it is. In addition, Physical functioning, Role lim physical, and Pain should correlate highest with the PCS and lowest with the MCS. Emotional wellbeing, Role lim emotional, and Social functioning should correlate highest with the MCS and lowest with the PCS. General health and Energy/fatigue should correlate moderately with both summary scores. In our data, Energy/fatigue correlates with the MCS stronger than it should, and with the PCS weaker than it should. **Pearson's correlation coefficient, continuous variables. ***Spearman's correlation coefficient, ordinal variables. P-values were adjusted using Benjamini-Hochberg correction where multiple testing was done. The following effect size criteria were chosen: < 0.3 weak; 0.3-0.5 moderate; > 0.5 strong.

Online Resource 4 The odds ratios (OR) of having better quality of life (QoL) score for patients with dry eye disease (DED) in comparison with those without DED according to three different DED diagnostic criteria (OSDI, DE, OSD).

	OSDI OR (95% CI)	DE OR (95% CI)	OSD OR (95% CI)
15D, N=574	**0.39 (0.26, 0.58)**	**0.37 (0.22, 0.64)**	**0.55 (0.40, 0.76)**
SF-36 PCS, N=424	0.73 (0.46, 1.14)	1.01 (0.56, 1.83)	0.71 (0.49, 1.04)
SF-36 MCS, N=424	**0.58 (0.38, 0.90)**	0.69 (0.38, 1.23)	0.69 (0.48, 1.00)
BDI-II, N=459	**0.50 (0.32, 0.79)**	**0.46 (0.25, 0.83)**	**0.57 (0.39, 0.84)**

QoL scores were stratified into ordinal categories based on the quartiles. The ORs were derived from the coefficients of the ordinal logistic regression, in which QoL scores were dependent variables. OR was adjusted for age, sex, BCVA and comorbidities, 95% confidence intervals are in the parentheses. Bolded ORs are significant based on their confidence intervals. The proportional odds assumption was tested with Brant-Wald test and no violation was detected. DED=dry eye disease, OSDI=ocular surface disease index, DE=dry eye, OSD=ocular surface disease, BCVA=best corrected visual acuity, CI=confidence interval, PCS=physical component summary, MCS=mental component summary, BDI=Beck's depression inventory. DED diagnostic criteria can be found in Table 3.

These results showed that in people with DED, the odds of having better QoL score were lower compared with people without DED with respect to 15D and BDI-II, regardless of the diagnostic criteria for DED used, and with respect to SF-36 MCS when the OSDI criterion is used. The ordinal logistic regression results are in keeping with the results of logistic regression presented in Table 4 and the conclusion, that DED imposes a significantly worse QoL.

Online Resource 5 Standardized Beta coefficients from the hierarchical linear regression investigating the association between DED signs and symptoms and quality of life measured with 15D, SF36 and BDI-II questionnaires*

	ALL SUBJECTS															
	15D INDEX				SF36 PCS				SF36 MCS				BDI-II			
	MODEL 1	MODEL 2	MODEL 3	MODEL 4	MODEL 1	MODEL 2	MODEL 3	MODEL 4	MODEL 1	MODEL 2	MODEL 3	MODEL 4	MODEL 1	MODEL 2	MODEL 3	MODEL 4
**Blepharitis**		-0.010	-0.009	-0.009		0.028	0.029	0.013		-0.062	-0.043	-0.039		0.010	0.010	0.017
**MGD**		-0.034	-0.024	-0.037		0.075	0.080	0.070		-0.055	-0.061	-0.059		0.079	0.071	0.081
**Conjunctival redness**		-0.015	-0.009	0.008		-0.091	-0.094	-0.079		-0.052	-0.054	-0.052		0.023	0.034	0.032
**BCVA**		0.016	0.031	0.057		-0.089	-0.084	-0.038		-0.096	-0.085	-0.096		0.040	0.036	0.035
**NIBUT**		0.041	0.033	0.023		0.047	0.038	0.028		0.052	0.054	0.048		0.010	0.010	0.012
**Schirmer**		-0.026	-0.019	-0.007		-0.051	-0.042	-0.037		0.000	0.009	0.036		0.006	-0.002	-0.035
**Corneal staining**		-0.057	-0.047	-0.052		0.029	0.028	0.022		-0.049	-0.039	-0.046		0.101	0.101	0.112
**Conjunctival staining**		-0.043	-0.032	-0.036		-0.055	-0.043	-0.035		-0.019	-0.016	0.004		0.113	0.109	0.089
**OSDI**	**-0.260**	**-0.253**	**-0.204**	**-0.194**	**-0.167**	**-0.177**	**-0.151**	**-0.125**	**-0.135**	**-0.154**	**-0.139**	-0.150	**0.170**	**0.174**	**0.160**	**0.165**
**Dryness subjective**			-0.109	-0.088			-0.096	-0.064			**-0.163**	-0.148			0.137	0.120
**DED med. freq.**			-0.058	-0.054			-0.016	-0.017			0.105	0.094			-0.094	-0.088
**Other ocular med. use**			0.070	0.068			0.102	0.099			-0.068	-0.055			-0.051	-0.072
**Cancer**				-0.086				-0.025				-0.044				**0.142**
**Connective tissue diseases**				-0.039				-0.066				0.005				0.027
**Diabetes**				**-0.112**				**-0.132**				-0.045				0.053
**Heart diseases**				-0.083				**-0.188**				0.114				0.085
**Hypertension**				**-0.128**				**-0.127**				-0.026				0.045
**Musculoskeletal conditions**				-0.007				-0.042				0.040				-0.044
**Psychiatric diseases**				-0.041				-0.091				-0.108				0.092
**Pulmonary diseases**				-0.086				-0.045				-0.078				0.029
**Vascular diseases**				-0.075				-0.032				0.024				-0.105
R^2	**0.17**	**0.18**	**0.21**	**0.30**	**0.12**	**0.14**	**0.16**	**0.30**	**0.05**	**0.08**	**0.11**	**0.14**	**0.10**	**0.14**	**0.15**	**0.20**
R^2 change		0.01	**0.02**	**0.10**		0.02	**0.02**	**0.13**		**0.04**	**0.02**	0.03		**0.04**	**0.02**	**0.05**
Adjusted R^2	**0.17**	**0.17**	**0.19**	**0.27**	**0.11**	**0.12**	**0.13**	**0.26**	**0.04**	**0.06**	**0.08**	**0.09**	**0.09**	**0.12**	**0.13**	**0.16**
Adjusted R^2 change		0.00	**0.02**	**0.09**		0.01	**0.01**	**0.12**		**0.02**	**0.02**	0.01		**0.03**	**0.01**	**0.04**

	WOMEN															
	15D INDEX				SF36 PCS				SF36 MCS				BDI-II			
	MODEL 1	MODEL 2	MODEL 3	MODEL 4	MODEL 1	MODEL 2	MODEL 3	MODEL 4	MODEL 1	MODEL 2	MODEL 3	MODEL 4	MODEL 1	MODEL 2	MODEL 3	MODEL 4
**Blepharitis**		-0.060	-0.053	-0.054		0.130	0.131	0.075		-0.088	-0.062	-0.037		-0.081	-0.089	-0.060
**MGD**		0.053	0.052	0.028		0.057	0.060	0.074		0.074	0.062	0.049		0.054	0.051	0.055
**Conjunctival redness**		0.017	0.034	0.053		-0.054	-0.044	-0.036		-0.040	-0.036	-0.058		0.024	0.026	0.054
**BCVA**		-0.019	-0.005	0.015		-0.146	-0.130	-0.088		-0.124	-0.120	-0.142		0.046	0.041	0.021
**NIBUT**		0.097	0.085	0.061		0.076	0.059	0.040		0.070	0.075	0.077		-0.001	-0.006	0.001
**Schirmer**		-0.022	-0.017	-0.009		-0.006	0.002	-0.014		0.024	0.030	0.063		0.014	0.010	-0.049
**Corneal staining**		-0.019	-0.003	-0.026		0.109	0.112	0.082		-0.071	-0.052	-0.058		0.071	0.066	0.088
**Conjunctival staining**		-0.061	-0.047	-0.063		-0.063	-0.054	-0.032		-0.031	-0.033	-0.028		**0.217**	**0.209**	0.166
**OSDI**	**-0.284**	**-0.276**	**-0.207**	**-0.232**	**-0.203**	**-0.235**	**-0.184**	**-0.208**	**-0.138**	-0.176	-0.165	-0.166	**0.188**	**0.186**	0.157	0.175
**Dryness subjective**			**-0.168**	-0.108			-0.141	-0.054			-0.084	-0.077			0.145	0.102
**DED med. freq.**			-0.044	-0.044			-0.033	-0.045			0.054	0.038			-0.052	-0.031
**Other ocular med. use**			0.069	0.052			0.088	0.094			-0.101	-0.092			-0.051	-0.092
**Cancer**				-0.051				-0.004				-0.018				0.112
**Connective tissue diseases**				-0.013				-0.073				0.053				0.038
**Diabetes**				**-0.171**				-0.131				-0.018				0.091
**Heart diseases**				-0.095				**-0.184**				0.107				0.111
**Hypertension**				**-0.172**				-0.057				-0.033				0.050
**Musculoskeletal conditions**				0.040				-0.007				0.064				-0.066
**Psychiatric diseases**				-0.025				-0.093				-0.059				0.131
**Pulmonary diseases**				-0.051				0.017				-0.083				-0.049
**Vascular diseases**				-0.119				-0.098				0.070				-0.145
R^2	**0.20**	**0.22**	**0.26**	**0.38**	**0.15**	**0.20**	**0.24**	**0.32**	**0.04**	**0.09**	**0.10**	0.13	**0.11**	**0.17**	**0.19**	**0.26**
R^2 change		0.02	**0.04**	**0.13**		**0.05**	**0.03**	**0.09**		0.04	0.01	0.03		**0.06**	0.02	**0.07**
Adjusted R^2	**0.19**	**0.19**	**0.22**	**0.34**	**0.14**	**0.17**	**0.19**	**0.25**	**0.04**	**0.05**	**0.05**	0.04	**0.10**	**0.14**	**0.14**	**0.18**
Adjusted R^2 change		0.00	**0.03**	**0.11**		**0.03**	**0.02**	**0.06**		0.01	0.00	-0.01		**0.04**	0.01	**0.04**

	MEN															
	15D INDEX				SF36 PCS				SF36 MCS				BDI-II			
	MODEL 1	MODEL 2	MODEL 3	MODEL 4	MODEL 1	MODEL 2	MODEL 3	MODEL 4	MODEL 1	MODEL 2	MODEL 3	MODEL 4	MODEL 1	MODEL 2	MODEL 3	MODEL 4
**Blepharitis**		0.074	0.076	0.081		-0.042	-0.042	-0.003		-0.012	0.011	-0.012		0.070	0.080	0.102
**MGD**		-0.140	-0.133	-0.117		0.067	0.050	0.050		-0.221	**-0.238**	-0.209		0.105	0.109	0.087
**Conjunctival redness**		-0.074	-0.084	-0.075		-0.150	-0.174	-0.168		-0.061	-0.105	-0.066		0.042	0.067	0.035
**BCVA**		0.055	0.070	0.082		-0.009	-0.014	0.024		-0.067	-0.046	-0.036		0.032	0.023	0.046
**NIBUT**		-0.032	-0.033	-0.036		0.036	0.026	0.011		0.038	0.025	0.006		0.022	0.029	0.022
**Schirmer**		-0.034	-0.029	-0.022		-0.117	-0.119	-0.105		-0.064	-0.057	-0.014		-0.014	-0.012	-0.023
**Corneal staining**		-0.121	-0.116	-0.118		-0.110	-0.120	-0.099		-0.027	-0.035	-0.010		0.134	0.136	0.126
**Conjunctival staining**		-0.019	-0.009	-0.011		-0.033	-0.031	-0.022		-0.008	0.006	0.015		-0.004	0.004	-0.015
**OSDI**	**-0.210**	**-0.199**	**-0.180**	-0.140	-0.075	-0.041	-0.046	0.040	-0.127	-0.129	-0.110	-0.103	**0.140**	0.136	0.134	0.121
**Dryness subjective**			-0.053	-0.053			-0.083	-0.095			**-0.272**	**-0.282**			0.135	0.136
**DED med. freq.**			-0.013	-0.009			0.116	0.090			0.203	0.193			-0.166	-0.171
**Other ocular med. use**			0.082	0.083			0.056	0.055			0.020	0.053			-0.045	-0.050
**Cancer**				-0.116				-0.077				-0.095				0.205
**Connective tissue diseases**				-0.087				-0.061				-0.031				-0.004
**Diabetes**				-0.048				-0.118				-0.111				0.026
**Heart diseases**				-0.046				-0.196				0.135				0.055
**Hypertension**				-0.074				-0.204				-0.008				0.011
**Musculoskeletal conditions**				-0.088				-0.066				0.077				-0.028
**Psychiatric diseases**				-0.009				-0.036				-0.150				0.051
**Pulmonary diseases**				-0.113				-0.125				-0.073				0.127
**Vascular diseases**				-0.039				0.006				-0.043				-0.051
R^2	**0.13**	**0.17**	**0.18**	**0.26**	**0.07**	**0.14**	**0.16**	**0.36**	0.03	**0.10**	**0.17**	**0.23**	**0.06**	**0.11**	**0.14**	**0.20**
R^2 change		0.04	0.01	**0.08**		**0.07**	0.01	**0.21**		0.07	**0.07**	0.06		0.05	0.03	0.06
Adjusted R^2	**0.13**	**0.14**	**0.14**	**0.19**	**0.06**	**0.09**	**0.09**	**0.28**	0.02	**0.05**	**0.10**	**0.12**	**0.05**	**0.07**	**0.08**	**0.10**
Adjusted R^2 change		0.01	0.00	**0.06**		**0.03**	0.00	**0.18**		0.03	**0.05**	0.02		0.01	0.02	0.02

* The table presents standardized Beta coefficients from hierarchical linear regression. Bold coefficients are statistically significant (adjusted p-value < 0.05). P-values were adjusted using Benjamini-Hochberg correction method. The table presents also R^2 and adjusted R^2 values and their change between consecutive models. Bold R^2 and adjusted R^2 values mean that the model's overall p-value was significant (< 0.05). Bold changes were statistically significant according to Anova analysis of the models (p-value < 0.05). Model 1 is adjusted for age, sex, and OSDI. Model 2 includes covariates in model 1 plus clinical signs of DED. Model 3 includes covariates in model 2 plus subjective dryness of the eyes, use of drugs for DED and other ocular medication. Model 4 includes covariates in model 3 and comorbidities (a full list of comorbidities in Online resource 1)
